# A Tournaisian (earliest Carboniferous) conglomerate-preserved non-marine faunal assemblage and its environmental and sedimentological context

**DOI:** 10.7717/peerj.5972

**Published:** 2019-01-03

**Authors:** Jennifer A. Clack, Carys E. Bennett, Sarah J. Davies, Andrew C. Scott, Janet E. Sherwin, Timothy R. Smithson

**Affiliations:** 1 Department of Zoology, University of Cambridge, Cambridge, UK; 2 School of Geography, Geology and Environment, University of Leicester, Leicester, UK; 3 Department of Earth Sciences, Royal Holloway University of London, Egham, UK

**Keywords:** Tetrapods, Rhizodonts, Dipnoans, Chondrichthyans, Charcoal, Micropaleontology, Sedimentology, Paleobotany, Paleoenvironments

## Abstract

A conglomerate bed from the Tournaisian Ballagan Formation of Scotland preserves a rich array of vertebrate and other nonmarine fossils providing an insight into the wider ecosystem and paleoenvironment that existed during this pivotal stage of Earth history. It challenges hypotheses of a long-lasting post-extinction trough following the end-Devonian extinction event. The fauna recovered includes a wide size range of tetrapods, rhizodonts, and dipnoans, from tiny juveniles or small-bodied taxa up to large adults, and more than one taxon of each group is likely. Some fauna, such as actinopterygians and chondrichthyans, are rare as macrofauna but are better represented in the microfossil assemblage. The fauna provides evidence of the largest Carboniferous lungfish ever found. The specimens are preserved in a localized, poorly-sorted conglomerate which was deposited in the deepest part of a river channel, the youngest of a group of channels. In addition to the fossils (micro- and macro-), the conglomerate includes locally-derived clasts of paleosols and other distinctive elements of the surrounding floodplains. Charcoal fragments represent small woody axes and possible larger trunk tissue from arborescent pteridosperms. Preservation of the fossils indicates some aerial exposure prior to transport, with abrasion from rolling. The findings presented here contrast with other published trends in vertebrate size that are used to interpret a reduction in maximum sizes during the Tournaisian. The richness of the fauna runs counter to the assumption of a depauperate nonmarine fauna following the end-Devonian Hangenberg event, and charcoal content highlights the occurrence of fire, with the requisite levels of atmospheric oxygen during that stage.

## Introduction

The 12–15 million years following the end-Devonian mass extinction, also known as the Hangenberg Event, was thought to show a depauperate postextinction trough for nonmarine vertebrates, especially tetrapods (Sallan & Coates, 2010). The hiatus in the tetrapod fossil record became known as “Romer’s Gap,” and covered the entire Tournaisian stage and much of the Viséan stage. Recently, studies of the Tournaisian stage Ballagan Formation in southern Scotland and northern England have cast doubt on that assessment (Smithson et al., 2012; Smithson, Richards & Clack, 2015; Clack et al., 2016; Clack, Porro & Bennett, 2018). Here, we present further evidence of the potential richness of the Tournaisian stage for our understanding of the evolution of life on land.

**Figure 1:**
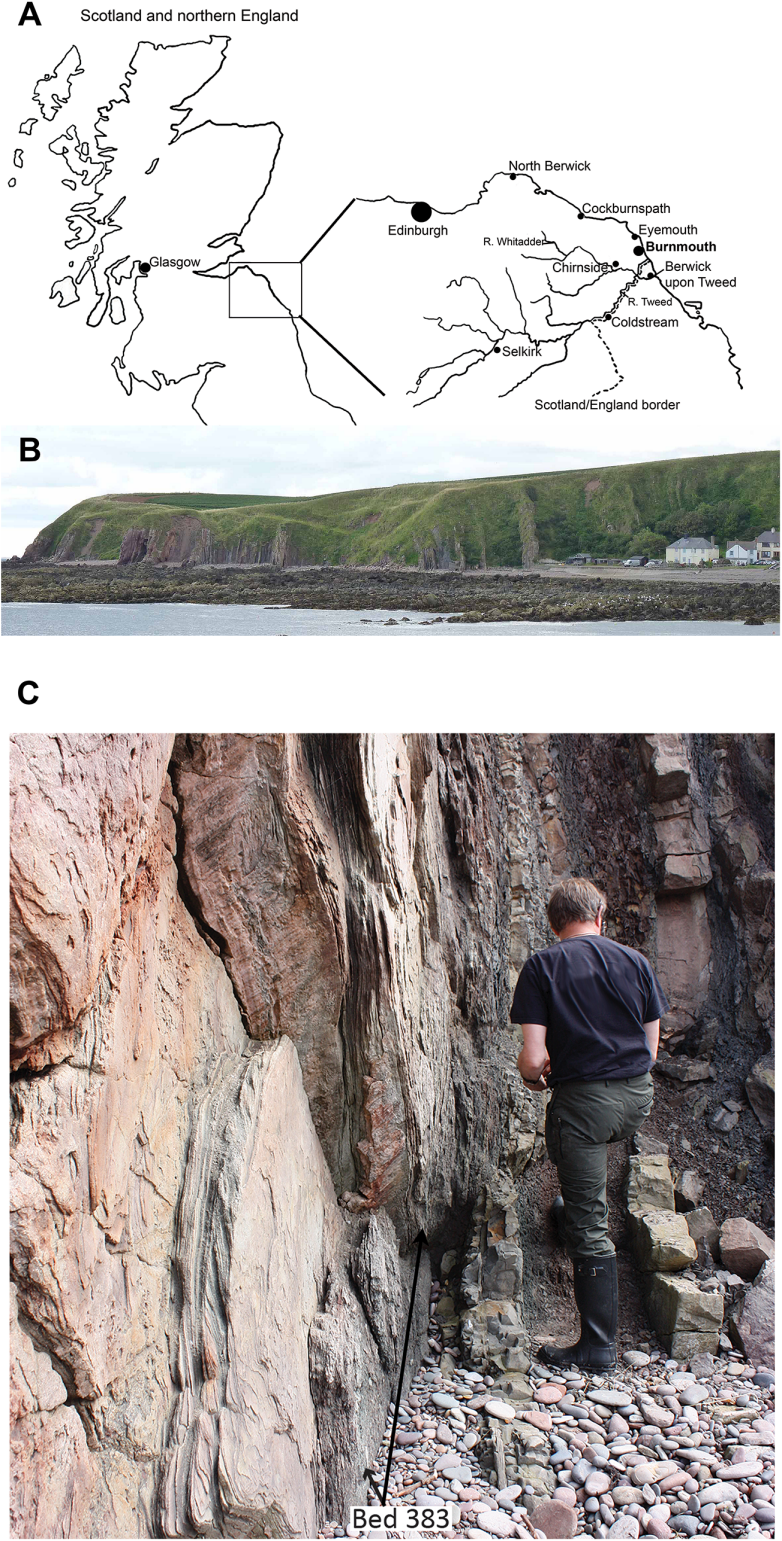
Geographical and geological context of Burnmouth and Bed 383. (A) A map of Scotland with inset showing locality of Burnmouth and major towns and rivers. (B) A view of the section exposed at Burnmouth. The horizon described lies close to the most prominent sandstone near the left-hand side of the photograph. (C) Photograph of the immediate region of Bed 383 prior to the 2018 rockfall, Rob Clack at the rock face (Photograph by SJD).

The coastal locality of Burnmouth, Scotland, about five miles (eight km) north of Berwick on Tweed, has long been known to geologists as part of the Berwick monocline (McAdam, Clarkson & Stone, 1992; Roper, 1997). The section at Burnmouth (Fig. 1) consists of strata that dip almost vertically along about a 500 m exposure through the mainly Tournaisian strata of the Ballagan Formation, formerly part of the Cementstone series in older terminology. Cementstones are also now known as dolostones.

The Devonian–Carboniferous boundary has been identified at the top of the Kinnesswood Formation near the base of the exposed succession (Millward et al., 2018a, 2018b; Marshall et al., in press) and the transition to the Viséan Fell Sandstones at its top. Its fossils have been little studied until recently. The geology was described for the British Geological Survey by Greig (1988, p. 49) who noted that plant fragments were common but fish fragments had only been found in two beds. McAdam, Clarkson & Stone (1992) also mentioned nonmarine invertebrates, fish spines, and plant remains in the Carboniferous section of Burnmouth. In a field guide to the locality, in part based on Grieg’s Memoir, Scrutton & Turner (1995) included a generalized geological map on which they numbered the large sandstone bodies 1–14 (1 being the youngest) that lie within the succession. They also noted that “fish fragments” had been found in the succession, but gave few details (Scrutton & Turner, 1995, p. 39 and 40).

In 1993, TRS began a more focused effort to examine Tournaisian rocks of Scotland for fossils. This formed part of an exploration of the Early Carboniferous in the Scottish Borders that had begun in 1988 at Coldstream (Smithson et al., 2012). Eventually, he discovered not only fish remains, but those of tetrapods. Subsequently, from 2006, more regular collecting was undertaken with the late Stan Wood, and in 2012, a NERC-funded consortium project focusing on the Tournaisian Ballagan Formation of Scotland, known as the TW:eed Project (Tetrapod World: early evolution and diversification), began an intensive exploration of Burnmouth and other Tournaisian localities. Several papers relating to the Burnmouth sediments and associated Tournaisian and other Early Carboniferous sites have already resulted from this project (Bennett et al., 2016; Clack et al., 2016; Coates & Teitjen 2018; Kearsey et al., 2016; Smithson, Richards & Clack, 2015; Smithson et al., 2017; Chen et al., 2018; Clack, Porro & Bennett, 2018; Clark, Miller & Ross, 2018; Millward et al., 2018a, 2018b; Richards et al., 2018; Ross et al., 2018; Smithson & Clack, 2018; Marshall et al., in press).

At least 11 beds with vertebrate bones including seven containing tetrapods (Clack et al., 2016) have been discovered in the Burnmouth succession so far. One particularly rich bed and its context is described by B.K.A. Otoo and members of the consortium team (Otoo, 2015; Otoo et al., 2018).

This account describes the faunal association of a single bed from macro- and micro-fossils, its floral association from plant remains, and sedimentological context from detailed studies of a conglomerate lens observed within the section at Burnmouth. Until recently, this was one of the best exposed and most accessible of the conglomerates present within the section at Burnmouth. It lies about 383 m above the Devonian–Carboniferous boundary (Clack et al., 2016; Clack, Porro & Bennett, 2018), as exposed at Burnmouth, henceforward referred to as Bed 383. Assuming, with caution, that the Tournaisian succession exposed at Burnmouth accumulated in proportion to the 12 million years of the stage, this would give an approximate date of 350 Ma for Bed 383, about nine Myr after the end of the Devonian. Other beds at Burnmouth mentioned here will be referred to by their heights above the D/C boundary as defined there. Unfortunately, exposure of and access to the Bed 383 was obliterated by a large rock fall between late March and early April 2018.

The fauna of Bed 383 includes numerous isolated bones, including both very large and very small examples, of tetrapods, lungfishes, rhizodonts, gyracanth spines and girdle elements, actinopterygians, a few teeth of the stem chondrichthyan *Ageleodus*, and chondrichthyan spines and scales. Many of the vertebrate specimens are fragmentary and indeterminate, while others can be identified to major clades or in a few cases more precisely. One tetrapod specimen is the partial lower jaw of a *Crassigyrinus*-like tetrapod (Clack, Porro & Bennett, 2018). The bed also contains charcoalified plant stems, charcoal fragments, and wood fragments. However, the more fragile ostracod and bivalve fossils that are a common element of sedimentary rocks are absent.

The conglomerate described is from a succession that has produced one of the richest assemblages of vertebrates from Burnmouth and in particular of tetrapods (Clack et al., 2016). It epitomizes the richness of Tournaisian vertebrate fauna and highlights the occurrence of fire, and the requisite levels of atmospheric oxygen during that stage. Among the aims of this paper is to draw attention to this richness, and its importance as a window into the terrestrial communities that existed in the earliest Carboniferous nonmarine environments. The existence of these specimens is important for inclusion in future paleobiology databases, to give a more accurate picture of life in the Early Carboniferous (= Mississippian).

## Materials and Methods

Collecting specimens presented difficulties as the beds at Burnmouth are almost vertically orientated and Bed 383 occurs between the overlying massive sandstone and an underlying dolostone, close to the cliff face (Fig. 1C). Losing important fragments into the gap between these two more competent beds generated by erosion of the relatively soft conglomerate was a problem. The entire collection was retrieved from a volume of no more than about a cubic meter, but because of the geological situation and with collections made on several separate occasions, no more accurate an estimate is possible. Many are no more than small fragments, but about half of them are identifiable to a major clade. Some specimens were collected individually from the bed, others were collected as bulk matrix which was disassembled in the laboratory. Most of the latter specimens were prepared manually from the heterogenous matrix with a mounted needle, dental mallet or air-pen. Thus, the list of specimens does not represent an exhaustive inventory of those elements present, nor should the collection of macrofossils be used in quantitative faunal analyses.

Sedimentological observations were made using a combination of detailed logging and tracing of surfaces and packages in the field at low tide, linked to an interpretation of outcrop photographs, satellite images, and “drone” footage. The “drone” footage was used to aid identification of key erosion surfaces and analyze the internal structures exposed at low tide across the wave cut platform. This detailed imagery was important for comparison with the aerial imagery (1 pixel = 10 × 10 cm) supplied by the North East Coastal Observatory that allows the architectural relationships of the exposed sandstone bodies to be interpreted without any distortion. Drone footage was taken with a DJI Phantom 2 Vision+ carrying a camera with a sensor size of 1.2/in, 14 megapixels, a resolution of 4,384 × 3,288, HD recording of 1080p30/1080i60, and a recording FoV of 110°/85°.

All specimens are housed in the University Museum of Zoology, Cambridge (prefixed UMZC). Most specimens have numbers with the formulae 2017.2.x and 2018.1.x. About half of those also with the earlier formula are from a lower bed (Bed 340.5) described by Otoo et al. (2018). Some specimens were registered from 2011 onward. There are about 600 registered specimens from this bed so far, but this is still in progress. Samples were collected by TRS from 1993 to 2007, and the rest collected by TW:eed team members from 2007 to 2017. Photographs of most macrofossil specimens were taken by JAC using a Panasonic Lumix DMC-LZ5 and processed using Adobe Photoshop CS suites 2017.1.1 release. Others were photographed by JAC or TRS with a Dino-lite Pro AM4000 digital microscope.

Some specimen-bearing samples were micro-CT scanned in the UMZC X-Tek scanner and segmented using Materialise’s Interactive Medical Image Control System (MIMICS R) Research v18 (biomedical.materialise.com/mimics; Materialise NV, Leuven, Belgium). Scan data: Tetrapod parasphenoid, Fig. 3E, 2017.2.187, Resolution/Voxel size 59.8178 μm, Filter one mm copper, kV 190, μA 160, Slices 1984; Tetrapod neural arch Fig. 4C, 2017.2.189g, Resolution/Voxel size 23.9724 μm, Filter one mm copper, kV 190, μA 160, Slices 1983; Tetrapod ulna Fig. 5C, 2017.2.51, Resolution/Voxel size 49.9248 μm, Filter None, kV 190, μA 175, Slices 1671.

For the micropaleontology, a sample weighing 55.74 g from the base of the bed was processed overnight in a 5% solution of H_2_O_2_. The residue was then wet sieved at 1,000, 425, 250, 125, 65 μm fractions and oven dried at 40 °C. All fossil specimens present were picked from the 1,000, 425, 250, and 125 μm fractions and total counts are recorded in SI4.

For the paleobotanical analysis, sediment samples around 10 g in weight were dissolved first in dilute HCl followed by concentrated HF in line with standard techniques (Scott, 2010; Glasspool & Scott, 2013). Charcoal fragments were picked under a binocular microscope and selected fragments were mounted on aluminum stubs and studied using a Hitachi S2400 SEM (Scott, 2010).

## Geological Context

The succession at Burnmouth covers most of the Tournaisian stage, somewhat condensed, but extending from slightly below the Devonian–Carboniferous boundary, located at the top of the red sandstone Kinnesswood Formation, through to the base of the Viséan Fell Sandstone. The bed is dated as within the CM (claviger–macra) palynozone, although the palynozonation of the Tournaisian stage in Scotland is being reviewed as a result of work undertaken as part of the TW:eed project (Marshall et al., in press).

**Figure 2:**
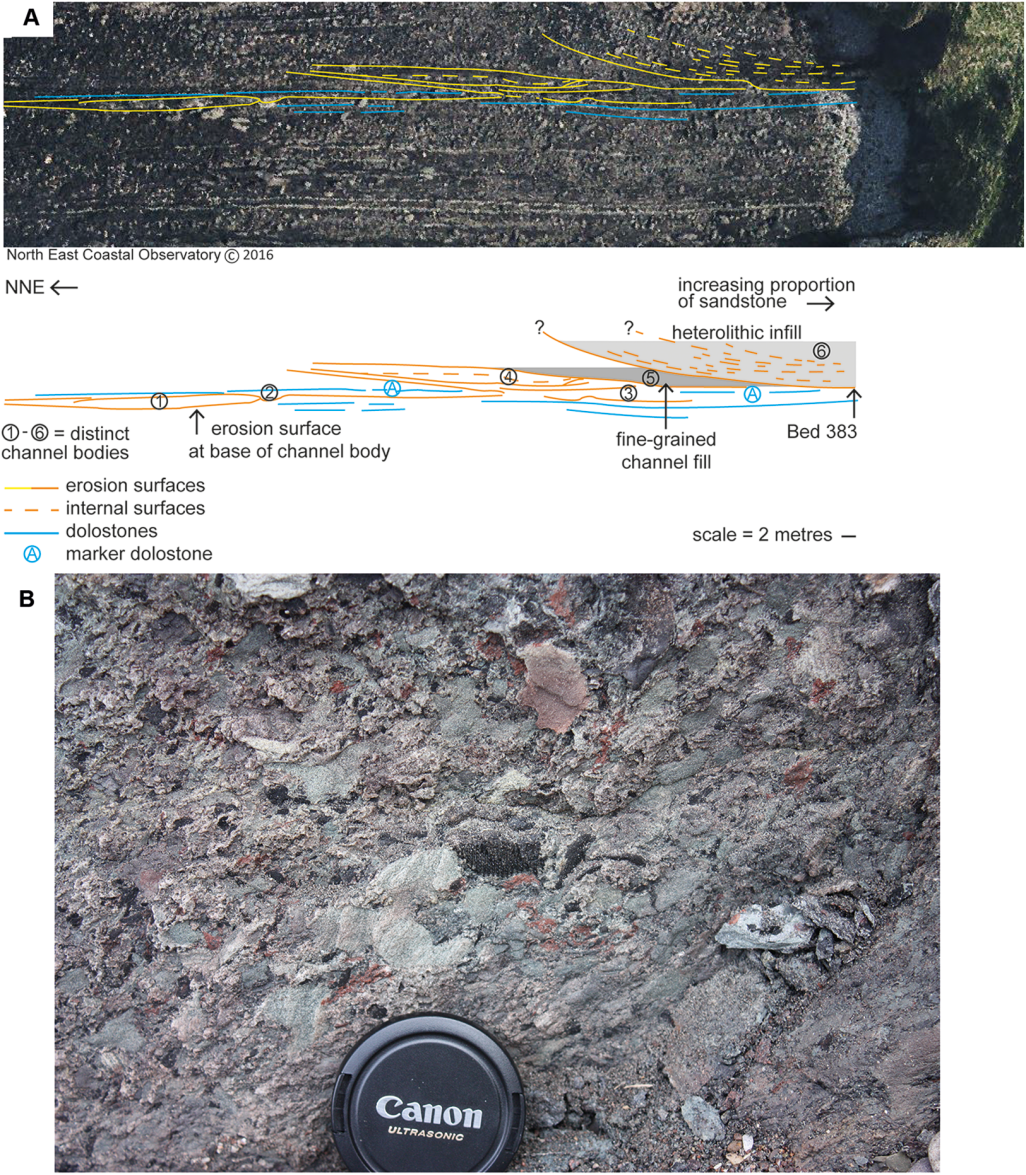
Geological context and channel interpretation. (A) Interpreted architecture of six distinct packages interpreted as river channel deposits. Bed 383 is located at the base of package 6 at the cliffward end barely extends onto the wave-cut platform. The erosion surface at the base of package 6 cuts down to a level below packages 4 and 5. The marker dolostone (A) can be traced into the cliff and is not removed by erosion associated with the base of package 6. (B) Close-up photograph of a sample of Bed 383 matrix.

The specimens are associated with a sandstone body ca. 383 m above the base of the Ballagan Formation at Burnmouth (Kearsey et al., 2016; Clack, Porro & Bennett, 2018). Analysis reveals six distinct sedimentary packages (Fig. 2A) that are exposed across the wave-cut platform at low tide and are broadly equivalent to sandstone 4 of Scrutton & Turner (1995). Packages 1–3 are ca. two m in thickness, and comprise fine- to medium-grained sandstones with slightly finer-grained tops. The bodies have basal erosion surfaces and shallow channelized forms. Package 1 is characterized by low-angle cross-stratification dipping north that passes into small-scale ripple cross-laminated structures as the body thins northward. Dolostone beds (including A on Fig. 2A and other overlying dolostones that are not illustrated separately due to imagery resolution) above packages 1–3 are truncated by the erosion surface at the base of package 4. This erosion surface has ca. four m of relief. Package 4 is sandstone-dominated with generally low-angle stratification, whereas package 5 is a gray, poorly-exposed siltstone that overlies an erosion surface that clearly truncates adjacent and underlying beds.

A red-brown, fine- to medium-grained sandstone, package 6, overlies the youngest erosion surface and is the thickest sandstone (minimum 6.5 m, Fig. 2A). The basal erosion surface cuts down to, but not through, dolostone beds (including A). *Chondrites* burrows occur in the dolostone beds underlying the sandstone succession at 383 m (Bennett et al., 2016). Package 6 (including Bed 383) is characterized by large-scale (broadly southerly) dipping stratification (depicted on Fig. 2). Cross-stratification (0.5 m scale) is observed between these surfaces particularly near the base. A 0.2 m-thick conglomerate occurs at the base of package 6 above this erosion surface and is now present only at the cliff though formerly it extended along the erosion surface onto the wave-cut platform exposures. This very localized conglomerate is poorly sorted, generally matrix supported, and variably cemented with dolomite (Fig. 2B). Centimeter-sized subrounded to angular clasts (typically up to 30 mm in length and elongate) comprise red and gray siltstone, organic material, and bioclasts, including the partial lower jaw (UMZC 2011.9.1, Clack, Porro & Bennett, 2018) and a range of microfossils. Clasts sit within a coarse-grained sandstone to granule-grade sandstone matrix, but clasts of the underlying dolostone are not identified in it. The specimens described in this paper derive from this conglomerate.

Plant material within the sediment comprises generally small fragments, both charred and uncharred, and is scattered within the matrix rather than occurring in layers. The charcoal samples comprise predominantly fragments one to three mm in length but larger pieces around five mm in length also occur. Better-preserved charcoal from a slightly lower bed (Bed 362) was used for comparison.

## Results

### Tetrapods: large elements Figs. 3–5

**Figure 3:**
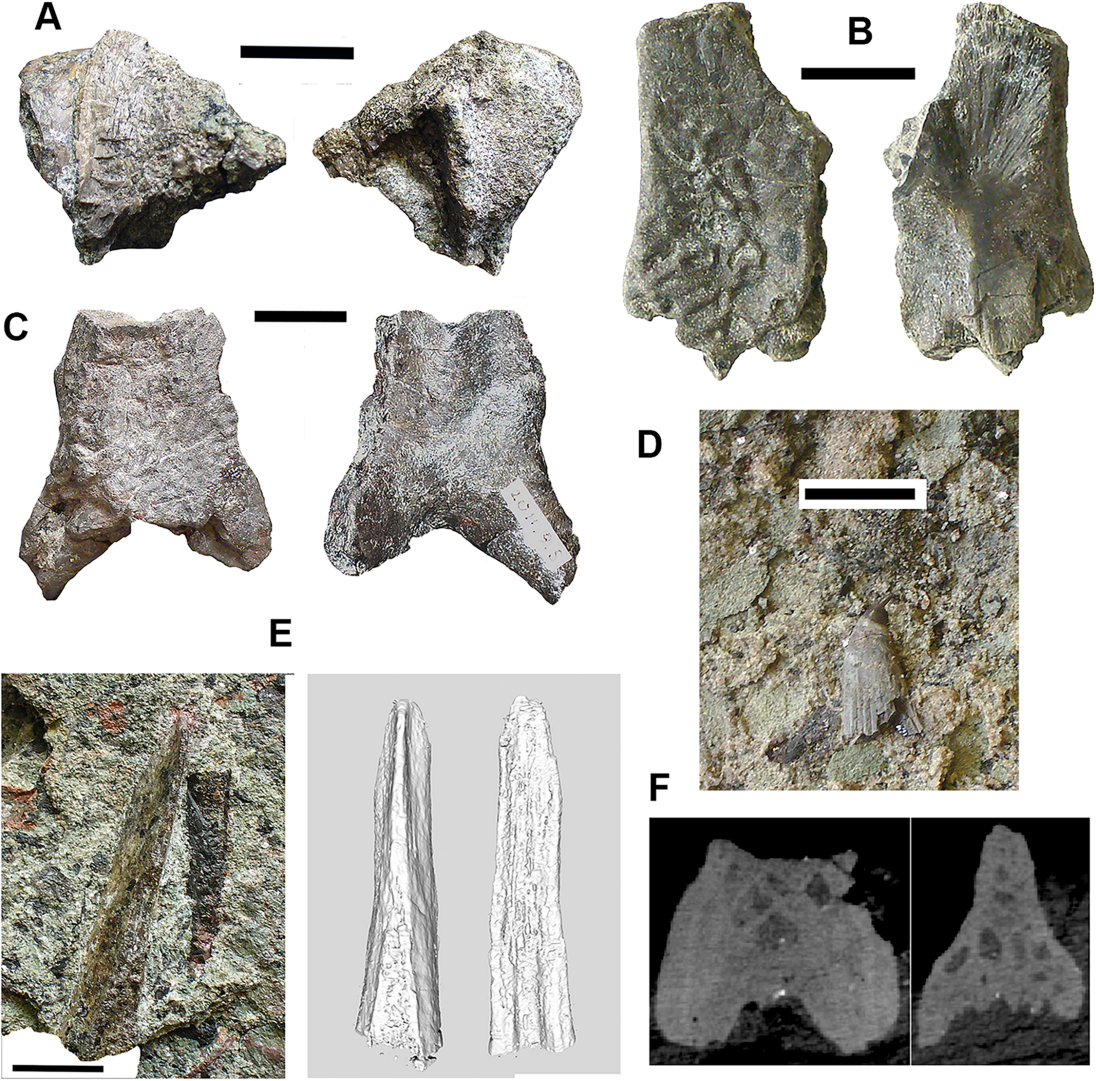
Large tetrapod skull bones. (A) Basipterygoid process 2017.2.12, dorsal view at left, ventral view at right. (B) Postfrontal 2017.2.545 dorsal view at left, ventral view at right. (C) Tabular 2011.9.5, dorsal view at left, ventral view at right. (D) Tooth 2017.2.384. (E) Parasphenoid 2017.2.187. Left image, photograph of the specimen, right image, micro-CT renderings, ventral view at left, dorsal view at right. (F) Parasphenoid 2017.2.187 micro-CT scan sections near base (left) and tip (right). All scale bars 10 mm.

*Braincase*: Two braincase elements have been identified. The anteriormost part of a parasphenoid cultrifom process (2017.2.187 Fig. 3E) of a large tetrapod shows a weakly denticulate ventral surface. The dorsal surface, seen from micro-CT scans (Fig. 3E), is strongly concave, and the body is pierced by canals and other foramina of uncertain function (Fig. 3F). The element is similar in size to that of *Crassigyrinus*, although the cultriform process in that animal has only a small oval area of denticulation further posteriorly (Panchen, 1985).

A basal process (2011.9.12 Fig. 3A) from the right side of the braincase is preserved with part of the basisphenoid. Grooves for the carotid arteries are clearly shown, as are the two facets of the basipterygoid process and its lining of dermal bone from the parasphenoid. Dorsally, a cavity probably represents part of the dorsum sellae. The element is large, and would indicate an individual larger than known *Crassigyrinus* specimens.

*Skull bones*: A robust but highly incomplete example of a tabular (2011.9.5 Fig. 3C) has worn but rugose dermal ornament on the dorsal surface. The posterolateral margin carries a broken tabular horn, grooved dorsally. The posterior margin carries a thin, narrow flange along its ventral edge, which presumably contacted an extension from the postparietal. The posterior margin curves strongly toward its medial edge, and with the postparietal would have formed a so-called “widow’s peak” as described in some embolomeres, but is more exaggerated in form. The bone is much larger than the equivalent in the known specimens of *Crassigyrinus*.

Identified as a tetrapod postfrontal, 2017.2.545 (Fig. 3B) has typically tetrapod pit and ridge dermal ornament. It is rather strongly developed in the posterior part of the bone, but lighter anteriorly. The bone is complete, with the orbital margin well defined, and sutural surfaces on each of the other sides. It has a sutural surface anteriorly for the prefrontal, and there is an excavation on the medial side for contact with the nasal. Medially is a contact for the parietal, and posteriorly two contacts, one presumably for the supratemporal and one more laterally, suggesting the presence of an intertemporal or a dorsal process of a postorbital. The bone relationships more closely resemble those of *Anthracosaurus* (Panchen, 1977) than embolomeres (Panchen, 1972) or *Eoherpeton* (Smithson, 1985).

*Tooth*: An isolated tooth (2017.2.384 Fig. 3D) is a blunt cone. It has labyrinthine infolding at the base, and an acuminate tip. It does not resemble that of a rhizodont tooth and is interpreted as tetrapod.

**Figure 4:**
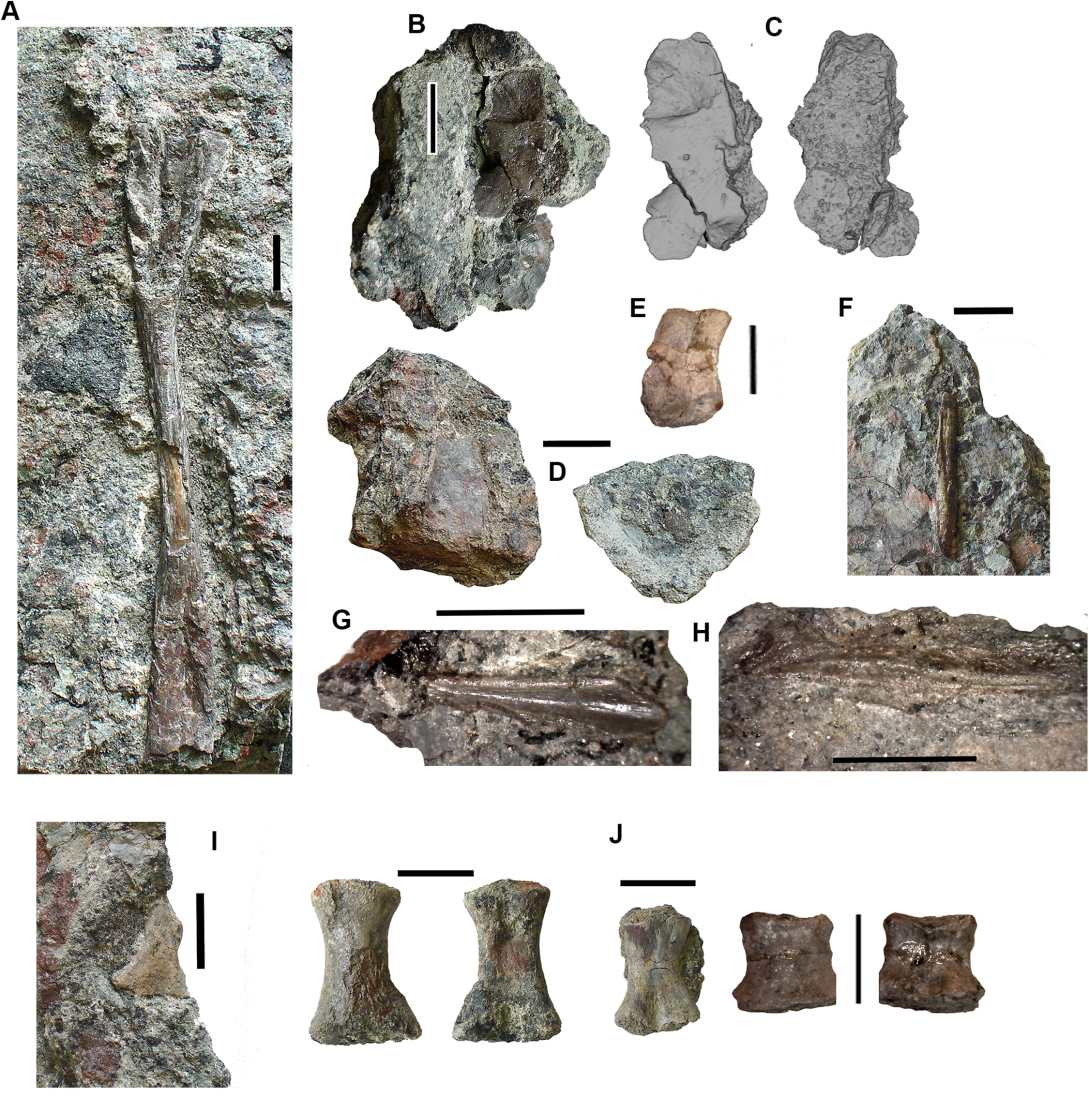
Large tetrapod postcranial bones. (A) Rib 2017.2.189a. (B) Neural arch 2017.2.189g. (C) Micro-CT scans of 2017.2.189g, external view at left, internal view at right. (D) Centra 2017.2.374, lateral view one centrum at left, anterior view of a second at right. (E) Small neural arch 2017.2.511. (F) Body scale 2017.2.107. (G) Body scale 2017.2.175. (H) Body scale 2017.2.144. (I) Phalanx 2017.2.61. (J) Metapodials or phalanges 2017.2.388, 2017.2.380, and 2017.2.61. All scale bars 10 mm.

*Postcranial elements, axial skeleton*: Centra, represented by two half-hoops (2017.2.374 Fig. 4D), show the typical morphology of gastrocentrous intercentra, similar in size to those known from *Crassigyrinus* (Panchen, 1985). The ventral and lateral surfaces are lined with smooth periosteal bone. Internally the surfaces are unfinished.

Neural arches are represented by one half of a neural arch (2017.2.189g Figs. 4B and 4C), probably an atlas arch somewhat similar to that of *Greererpeton* (Godfrey, 1989), and 2017.2.511, half of a neural arch (Fig. 4E). Specimen 2017.2.189g has little remaining evidence of zygapophyses, and the neural spine is thin with the distal portion somewhat compressed, leaving a transverse ridge across its base. Micro-CT scans show the mesial surface of unfinished bone where it adjoined its antimere and the underlying centra. An atlas arch of this size probably belonged to a moderately large individual, and it was found associated with the *Crassigyrinus*-like rib 2017.2.189a.

Several large ribs or parts of ribs most closely resemble those of *Crassigyrinus*. A large example (2017.2.189a Fig. 4A) has a striated surface. Its ends are somewhat crushed, but this seems to result purely from compression that did not exaggerate the expansion of both ends. In the figure, the probable proximal end is to the top. There is no apparent curvature, but that is usual for cervical ribs in early tetrapods including *Crassigyrinus*, as is the expanded distal end (Panchen, 1985). Rib 2017.2.49 (not figured) is of a similar size, but has lost both ends and is straight. The striation resembles that of 2017.2.189a, but identity as a lungfish rib is not ruled out.

*Postcranial elements, appendicular skeleton*: Several metapodials and phalanges including an ungual have been identified. A slightly asymmetrical autopodial bone (2017.2.388 Fig. 4J, left pair) is smooth and convex in dorsal view, and concave and grooved medially in ventral view. Others include 2017.2.380 (Fig. 4J, central image) exposed in ventral view only. It is more symmetrical but is also grooved medially. A shorter element, 2017.2.61, is probably a phalanx (Fig. 4J right pair). A probable ungual (2017.2.382) is exposed in dorsal view (Fig. 4I). It is flattened with an expanded proximal end and a rounded distal end. It most closely resembles the unguals of *Greererpeton* (Godfrey, 1989).

Narrow body scales with longitudinal grooves and ridges very similar to those of *Crassigyrinus* specimens (Panchen, 1985) have been found: 2017.2.107, 2017.2.175, and 2017.2.144 (Figs. 4F, 4G and 4H).

### Tetrapods: small elements

Alongside the large elements are a few moderate sized and much smaller bones, showing the presence either of small-bodied individuals or juveniles. Delicate ribs, limb bones, and small phalanges have been found.

*Skull and jaw bones:* An almost complete jugal (2016.13 Clack et al., 2016, fig. 6c) is exposed in internal view. The postorbital region is relatively long, the orbit margin moderately deep, and the preorbital region expands slightly anteriorly. The internal view shows no evidence of a lateral line. In terms of its orbital component it falls somewhere between the shallow orbit of *Ossirarus* and the deep orbit with a narrow suborbital bar in *Diploradus* (Clack et al., 2016), and more closely resembles that of *Whatcheeria* in proportion (Lombard & Bolt, 1995; Daeschler, Clack & Shubin, 2009) although this jugal is from a much smaller individual. Whatcheeriid-like remains have been found elsewhere in the Burnmouth sequence (Clack et al., 2016; Otoo, 2015; Otoo et al., 2018).

**Figure 5:**
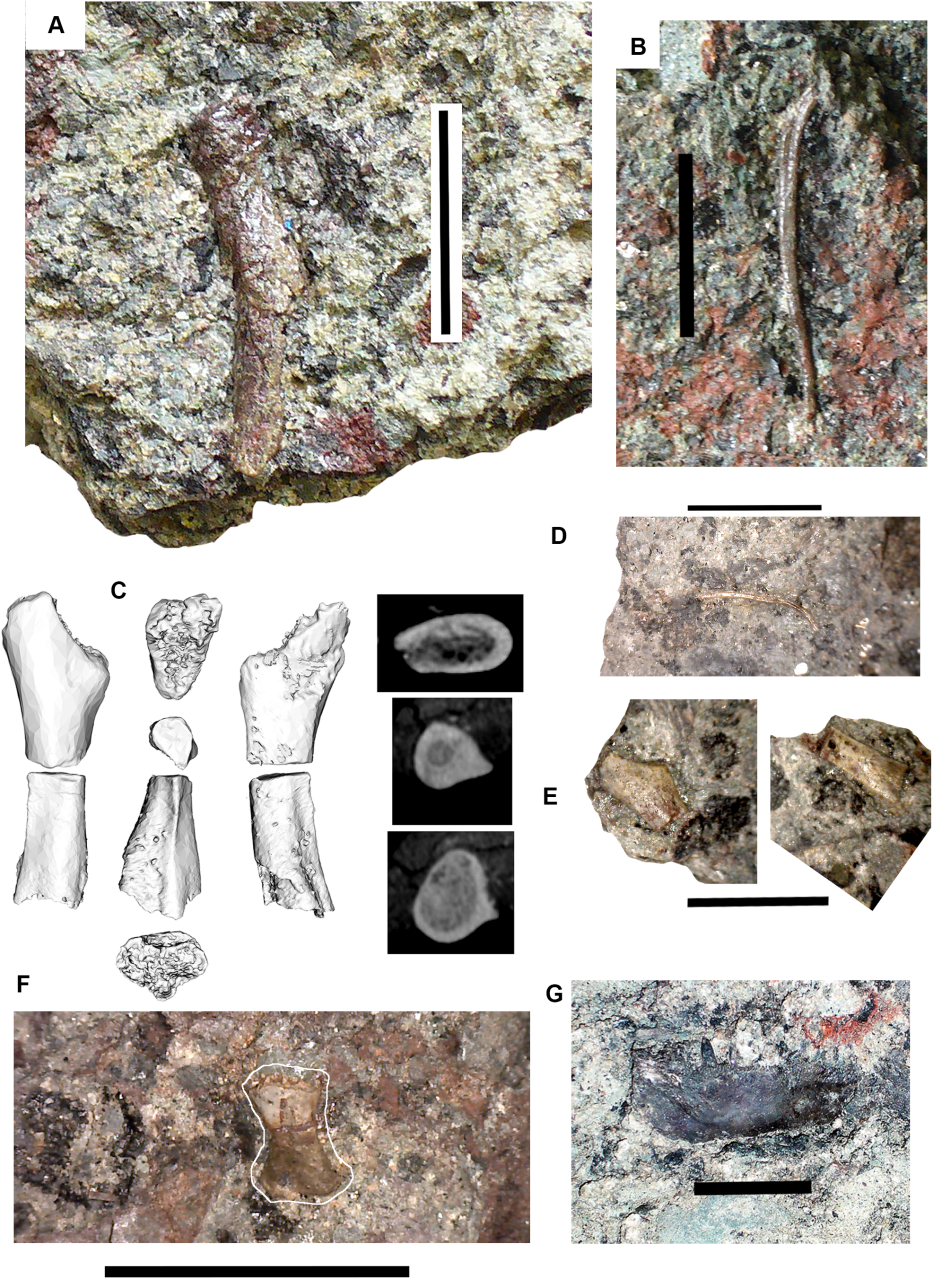
Small tetrapod elements. Small tetrapod elements. (A) Rib 2017.2.476. (B) Rib 2017.2.170. (C) Ulna 2017.2.51 from micro-CT scan. Views of the sections at right with the top image from near the proximal end, the middle image from mid-shaft and the lower image from near the distal end. (D) Rib 2017.2.149. (E) Ulna 2017.2.51 in part and counterpart, proximal end at left. (F) Ungual (outlined in white) 2017.2.382. (G) Jaw 2017.2.443. All scale bars 10 mm.

Two small partial jaws are interpreted as tetrapod on the basis of tooth shape and surface ornament. Neither the smaller, 2017.2.117 (not figured) nor the larger, 2017.2.443 (Fig. 5G) is identifiable to a higher taxon, but with other smaller elements, they show the presence of small individuals.

*Postcranial elements*: A small tetrapod ulna 2017.2.51 (Figs. 5C and 5E) is preserved in two counterpart pieces that have been micro-CT scanned. It shows a moderate olecranon process and a thick cortex at mid-shaft.

Small, and some very small, ribs have been identified. A small almost parallel-sided rib 2017.2.476 (Fig. 5A) has an incipient uncinate process. Ribs 2017.2.149 (Fig. 5D) and 2017.2.170 are extremely slender and curved. 2017.2.170 (Fig. 5B) is more strongly curved at its thicker and presumed proximal end, which is also grooved and has a slight sigmoid bend at its distal end. The rib head is not preserved.

### Lungfish Figs. 6–10

**Figure 6:**
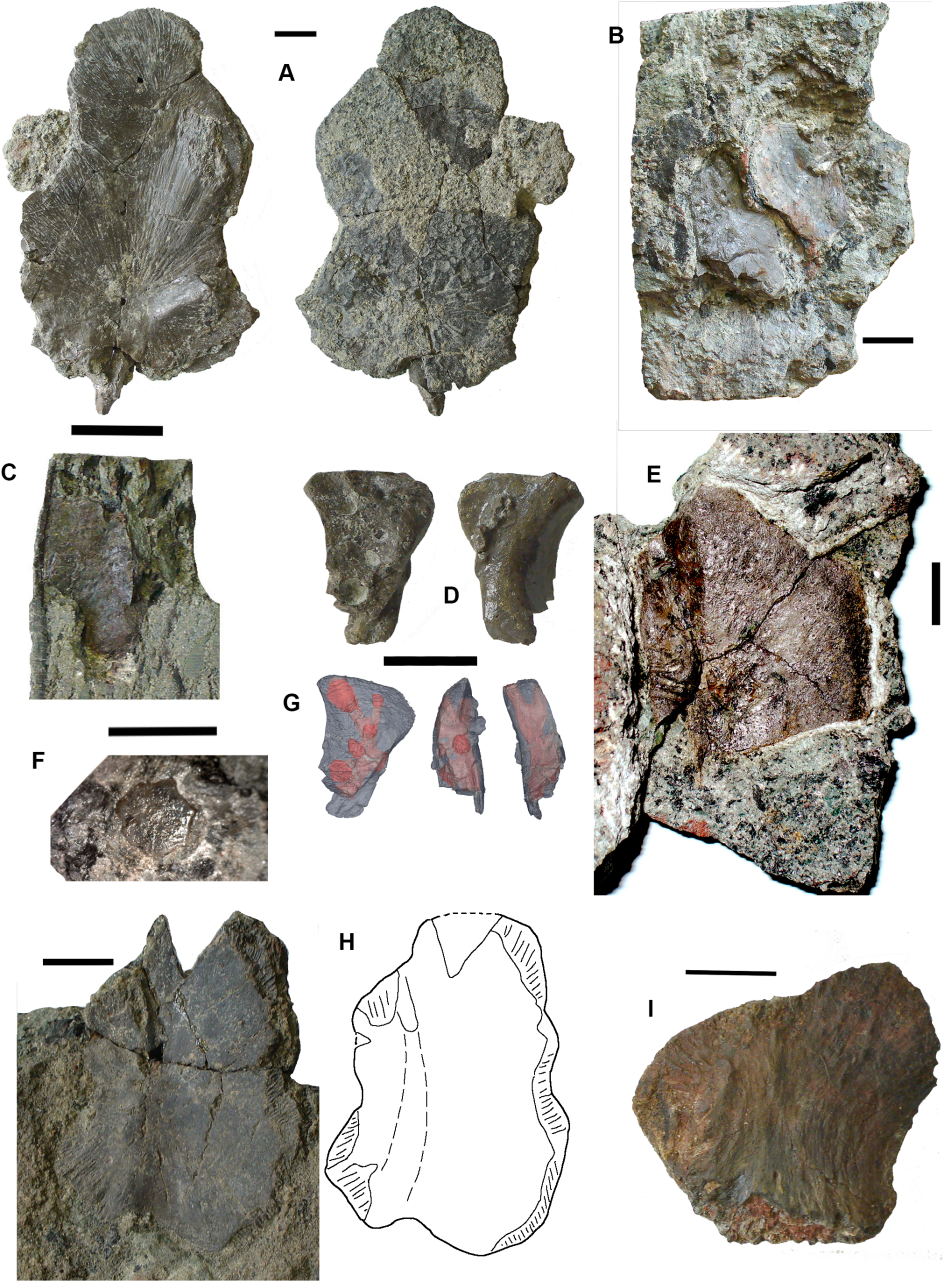
Lungfish skull bones. (A) Conjoined J- and I-bones. Ventral view at left, dorsal view at right. (B) Skull bone 2017.2.722, lying partially covered by a rhizodont scale. (C) Skull bone 2017.2189i. (D) Circumorbital bone 2011.9.7, dorsal view at left, ventral view at right. (E) Skull bone 2011.9.10. (F) Skull bone 2017.2.193. (G) Micro-CT renderings showing lateral line canals. (H) Skull bone 2017.2.443. (I) Skull bone 2018.1.2. All scale bars 10 mm.

*Skull bones*: Conjoined J- and I- skull bones 2017.2.405 (Fig. 6A) have rugose ornament of pits and ridges externally, but the strongly adhering matrix does not allow the lateral lines to be traced. The I-bone has a posterior process, which would have articulated with an anocleithrum. Internally, the centers of growth can be seen from the direction of the striations. The sutures between the bones are almost fused and difficult to trace in places. These bones would represent the skull of a very large individual.

A large robust skull bone 2017.2.550 (Fig. 6H) is visible mainly in internal view. The bone is complete except for wedge-shaped gaps at two places in the margin. Regions of sutural overlap surface mark the edges indicating the contact with at least four different bones. The possible course of a lateral line is indicated at one end by a groove and for the rest of its path, a low smooth ridge. Its shape is comparable with the KL-bone of some *Ctenodus* specimens (Sharp & Clack, 2013). The small area of exposed external surface shows low irregular ornament.

A moderately large bone (2018.1.2 Fig. 6I) has an irregular hexagonal shape with sutural overlap areas on two sides, and an unsutured concave margin on another. It is identified as lungfish on the lack of dermal ornamentation. A probable lungfish skull bone 2017.2.722 (Fig. 6B) has a smooth surface and lateral line pits. It is a long oval with an overlap surface partly broken off. A rhizodont scale is adjacent. It is not possible to identify the skull bone precisely within the skull. Skull bone 2011.9.10 (Fig. 6E) is an irregular hexagon in internal view, with a more or less smooth surface, whereas skull bone 2017.2.189i (Fig. 6C) is an oval bone with a plane surface, no strong ornament, but marked with small pits. Numerous small skull bones are preserved, mainly hexagonal or somewhat elongated with smooth surfaces and simple straight or curved margins, exemplified by 2018.2.193 (Fig. 6F).

**Figure 7:**
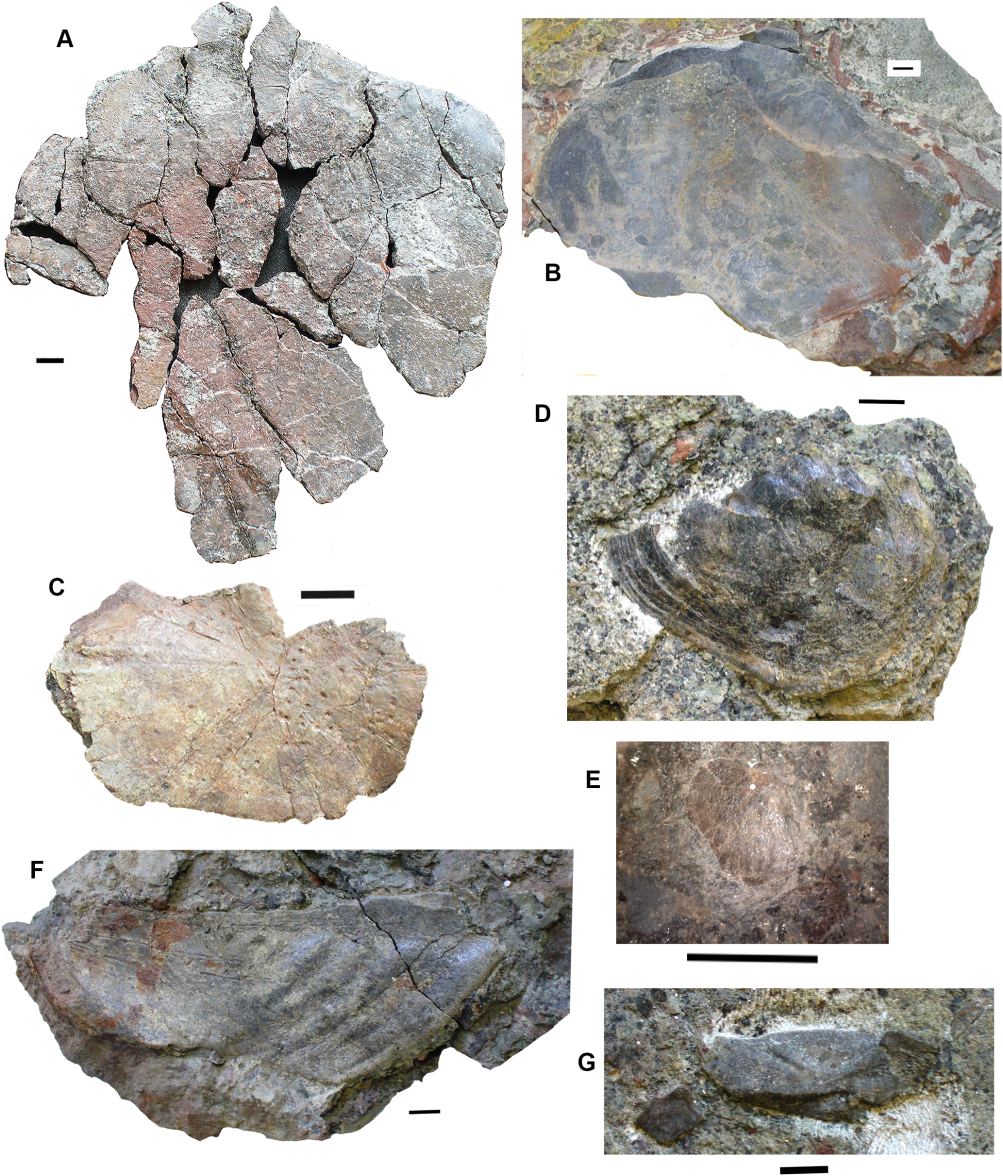
Lungfish opercular series. (A) Operculum 2017.2.188. (B) Operculum 2017.2.667. (C) Operculum 2011.9.6. (D) Operculum 2018.1.5. (E) Operculum 2017.2.720. (F) Suboperculum 2018.1.7. (G) Possible suboperculum or submandibular 2018.1.5. All scale bars 10 mm.

Bones 2011.9.7 (Fig. 6D), 2017.2.68, 2017.2.53, and 2017.2.547 (latter three not figured) are all probably circumorbital bones, the first three from a large individual. 2011.9.7 shows lateral line pores on the surface and has been micro-CT scanned (Fig. 6G) to reveal the course of the branches through the bone. All three bones are extremely robust. 2017.2.547 is more slender, elongated, and curved, showing two lateral line pores. *Opercular series*: Two very large opercula, 2017.2.188 (Fig. 7A) and 2017.2.667 (Fig. 7B) roughly similar in size, both appear to represent the same side of an animal, so represent at least two large individuals. The more complete 2017.2.188 is hexagonal in shape, with a small dorsally placed projection. Specimen 2017.2.667 was collected as an in situ block positioned further seaward in the bed than most of the other specimens, in a part of the bed that was subsequently completely removed by erosion. Its matrix is more coherent with fewer inclusions than seen in the more cliff-ward section, and may indicate one margin of the fossiliferous channel-fill. Potentially, the J + I-bones and the circumorbitals are from an individual of a similar size to those relating to the opercula. Smaller examples include 2018.1.5 (Fig. 7D) and 2011.9.6 (Fig.7C). The former is more or less circular, whereas the latter is an elongate hexagon in shape. Alternatively, that may be a suboperculum. A very small example 2017.2.720 (Fig. 7E) has a slightly striated surface.

A large suboperculum 2018.1.7 (Fig. 7F) is of a size commensurate with those of the large operculars and 2018.1.5 (Fig. 7G), and 2017.2.543 (not figured) are parts of bones that may be either subopercula or submandibulars.

**Figure 8:**
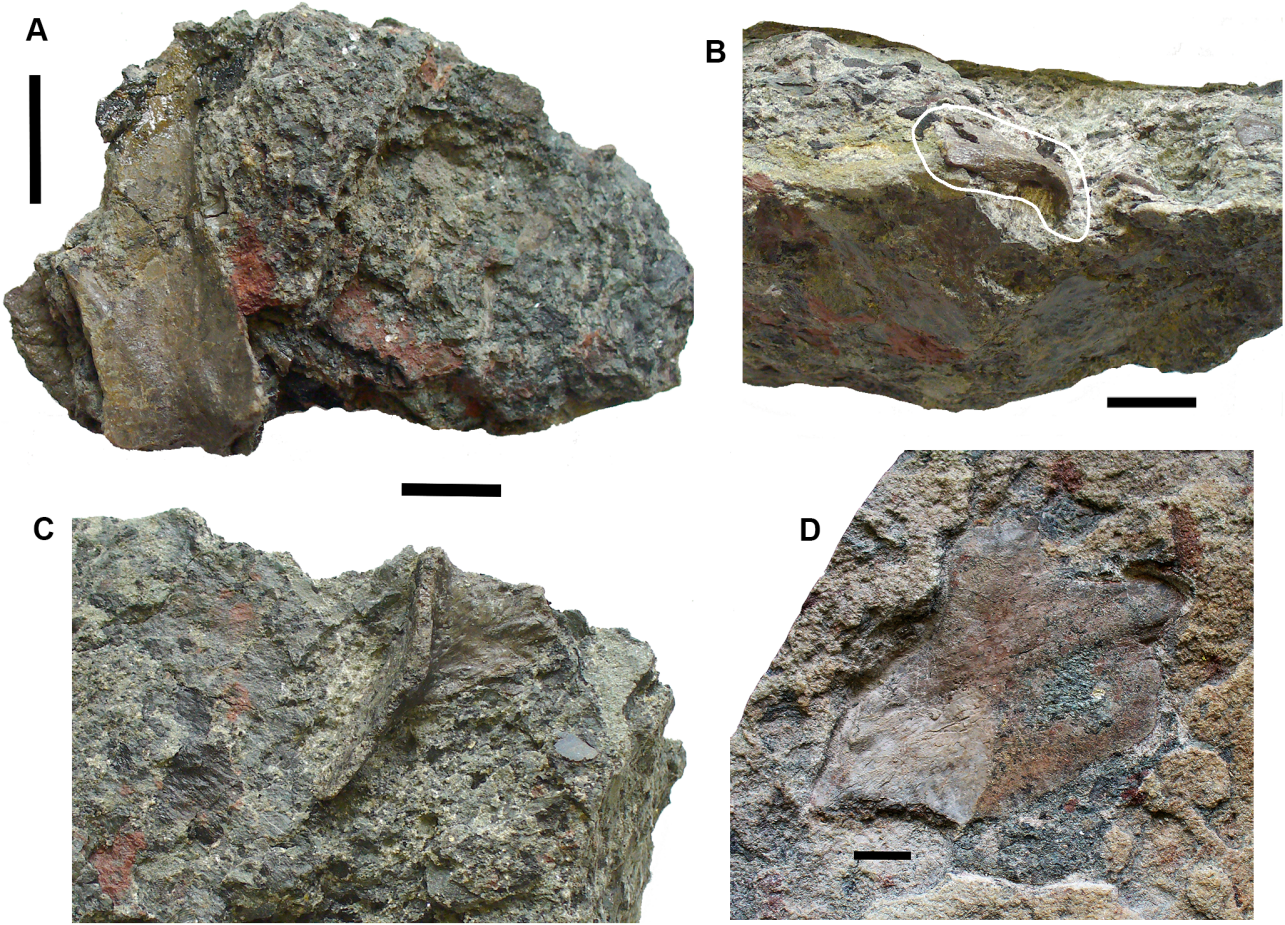
Lungfish shoulder girdle elements. (A) Partial clavicle 2017.2.70. (B) Partial cleithrum 2017.2.494a. (C) Possible central element 2017.2.398. (D) Anocleithrum 2017.2.57.

*Shoulder girdle*: Several elements of lungfish shoulder girdles are present, although only the anocleithrum is well represented. The best preserved is 2017.2.57 (Fig. 8D), a large example. It is a flattish bone bearing two obvious processes, but is otherwise featureless. The broader of the two processes is assumed to be that which contacted the I-bone. The other has not been entirely exposed but is narrower and more tapered. The presumed external surface onto which the cleithrum would have fitted is not visible. It could have belonged to similarly-sized individuals as did the large opercula. Other smaller and incomplete examples are in the collection.

Partial clavicles and cleithra include 2017.2.70 (Fig. 8A), a partial clavicle and 2017.2.546 (not figured), the distal end of a clavicle. The clavicle is curved in cross section and probably represents the lower part of the bone, lacking the ventral and distal sections. Specimen 2017.2.546 (not figured) shows the rugose sutural surface for the cleithrum, with the external face showing low pustulate ornament. Alternatively, it may be the ventral end of the cleithrum. A small cleithrum is represented by 2017.2.494a (Fig. 8B).

A possible centrum element is 2017.2.398 (Fig. 8C). This bone consists of a shallow curved part that bears a grooved and ridged flange at right angles to it. A similar bone was identified by the late Mahala Andrews (T.R.S. Andrews, 1979, personal communication) from the Serpukhovian of the Dora Bone Bed near Cowdenbeath (see Smithson, 1985, for details of the excavation there). It does not resemble any element preserved on the almost complete postcranial skeleton of *Ctenodus interruptus* (Sharp & Clack, 2013), so its identity remains in doubt.

**Figure 9:**
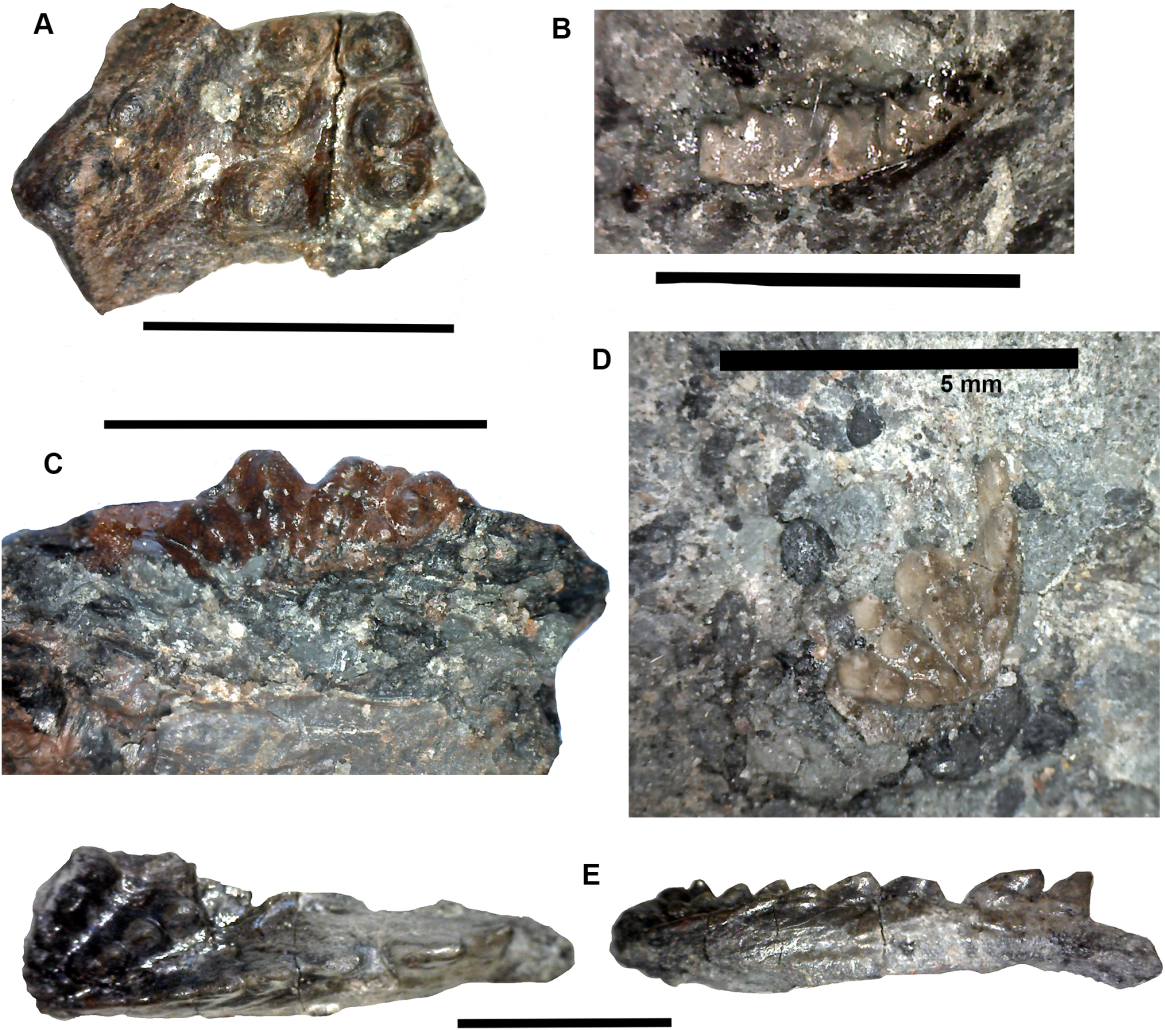
Lungfish tooth plates. (A) 2017.2.54. (B) c.f *Ballagadus caustrimi* 2017.2.66. (C) *B. caustrimi* 2017.2.67. (D) *B. caustrimi* 2017.2.513. (E) *Ballagadus rossi* 2014.1.2, buccal view at left, lingual view at right. All scale bars except (D) 10 mm, (D) 5 mm.

*Tooth plates*: Five lungfish tooth plates are present. These include two incomplete specimens representing tooth plates approximately 24 mm long and three much smaller tooth plates up to 3.5 mm long. One of the largest specimens, 2014.1.2 (Fig. 9E) was referred to *Ballagadus rossi* (Smithson, Richards & Clack, 2015). It consists of a complete tooth ridge 1 and partial tooth ridges 2 and 3 from a right pterygoid tooth plate. Ridge 1 is 24 mm long and bears eight laterally compressed teeth. The six oldest teeth are worn to a narrow blade. Teeth seven and eight show little wear, are curved slightly anteriorly and separated from the older teeth by a distinct gap. The teeth on ridges 2 and 3 are longitudinally compressed. The second of the larger incomplete specimens 2017.2.54 (Fig. 9A) consists of three incomplete rows of teeth representing parts of ridges 1, 2, and 3. The teeth are similar in size and shape to those on 2014.1.2 with anteriorly curved, laterally compressed teeth on ridge 1 and longitudinally compressed teeth on ridges 2 and 3. The teeth are relatively large and probably formed part of the labial edge of the plate.

The three smaller tooth plates (Figs. 9B, 9C and 9D) are of similar size and morphology. The most complete is 2017.2.513 (Fig. 9D). It is approximately triangular-shaped, 3.5 mm long and 2.5 mm wide with a length to width ratio of 1.4:1. There are four tooth ridges radiating from a point immediately behind the posterior end of tooth ridge 1. The tooth ridge angle is approximately 80°. Ridge 1 has four laterally compressed teeth, ridges 2 and 3 have four longitudinally compressed teeth and ridge 4 has three. 2107.2.67 (Fig. 9C) is missing many of the labial teeth but has five ridges and the tooth ridge angle is approximately 110°. These smaller tooth plates are similar to those of *Ballagadus caustrimi* (Smithson, Richards & Clack, 2015) which have been found in two older beds at Burnmouth (Bed 333 and Bed 340.5).

### Rhizodonts Figs. 10–15

**Figure 10:**
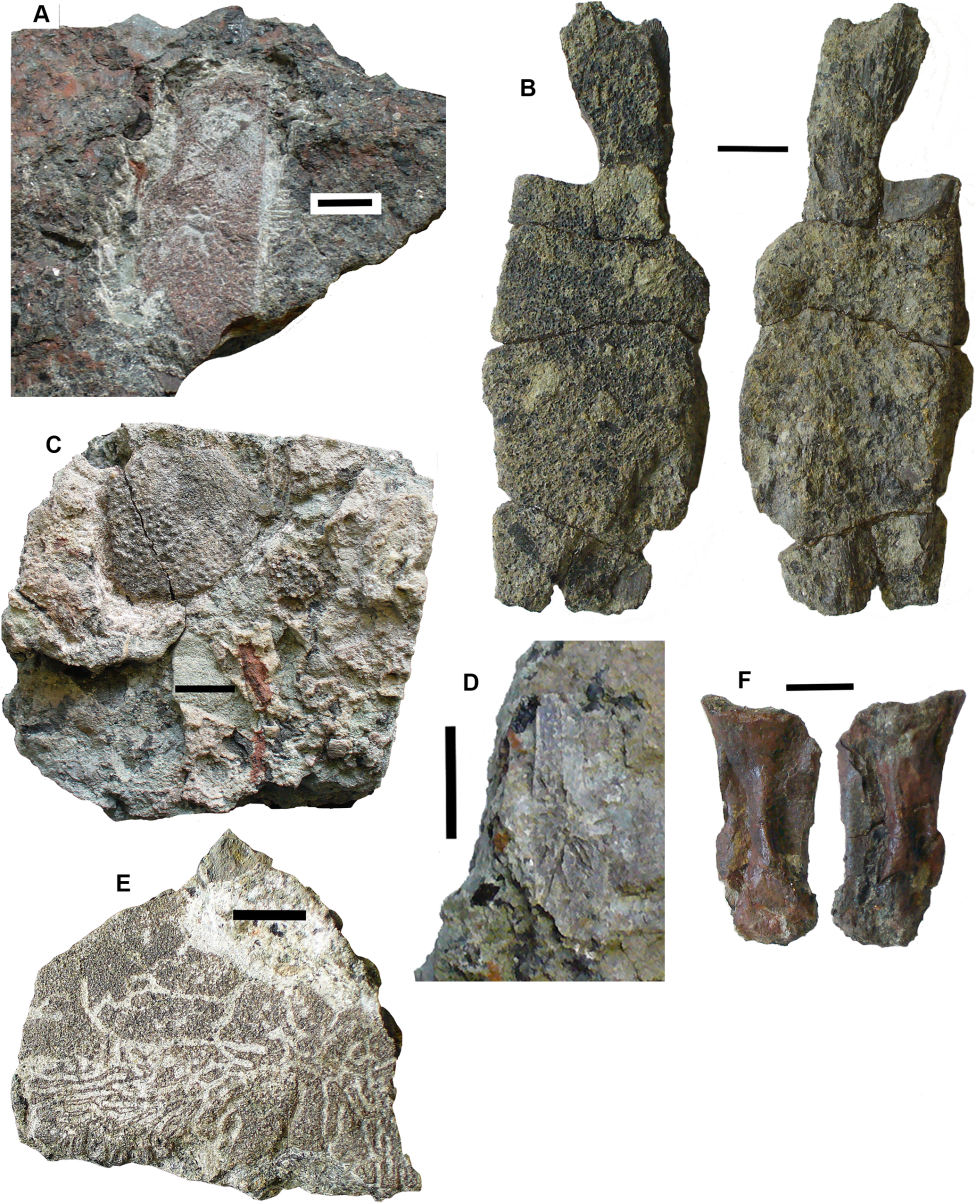
Rhizodont skull bones and hyomandibula. (A) Postparietal 2017.2.186. (B) Postparietal 2017.2.406. External view at right, internal view at left. (C) Extrascapula 2017.2.94. (D) Postparietal 2017.2.643. (E) Possible partial postparietal 2017.2.52. (F) Hyomandibula 2017.2.338. All scale bars 10 mm.

*Skull roof*: Postparietals include 2017.2.406 (Fig. 10B), which has part of its medial edge broken but is otherwise similar in shape to that of *Strepsodus* (Andrews, 1985; Jeffery, 1999). It has the typical pustulate ornament externally as found in *Strepsodus* in this and other beds at Burnmouth. Others include 2017.2.186 (Fig. 10A), a postparietal with overall fine pustulate texture externally, but with irregular grooved vermiform channels in which it is unlike that of 2017.2.406 or that of *Strepsodus* or *Screbinodus*. A probable partial postparietal, 2017.2.52 (Fig. 10E) has similar surface texture to 2017.2.186, particularly on the longer, left side. The internal surface shows a raised ridge. It is broader and shorter than that in 2017.2.406. A very small postparietal, 2017.2.643 (Fig. 10D), shows similar vermiform ornament invading a pustulate base, as 2017.2.186 and 2017.2.52, suggesting that this form of ornament is not the result of erosion or a feature of older individuals. It is narrow with a straight edge, presumed to be the medial edge, forming the midline. The lateral edge is somewhat broken, but may be a juvenile of that seen in Fig. 10D. An extrascapula, 2017.2.94 (Fig. 10C), has tuberculate ornament which is eroded away on the right-hand side.

The stout hyomandibular bone, 2017.2.338 (Fig. 10F), has a proximal articulation at an angle of about 45° to the long axis of the bone. It appears relatively short compared to that of a tristichopterid such as *Eusthenopteron* (Jarvik, 1980) and there is no sign of a foramen for the chorda tympani. Although compressed, the bone shows a prominent longitudinal ridge down one side, and a groove, not quite opposite it, on the other. It is most similar to that of GLAHM (Glasgow Hunterian Museum) V8038 illustrated by Jeffery (1999) except that in the latter the proximal end appears incomplete and its distal end wider.

**Figure 11:**
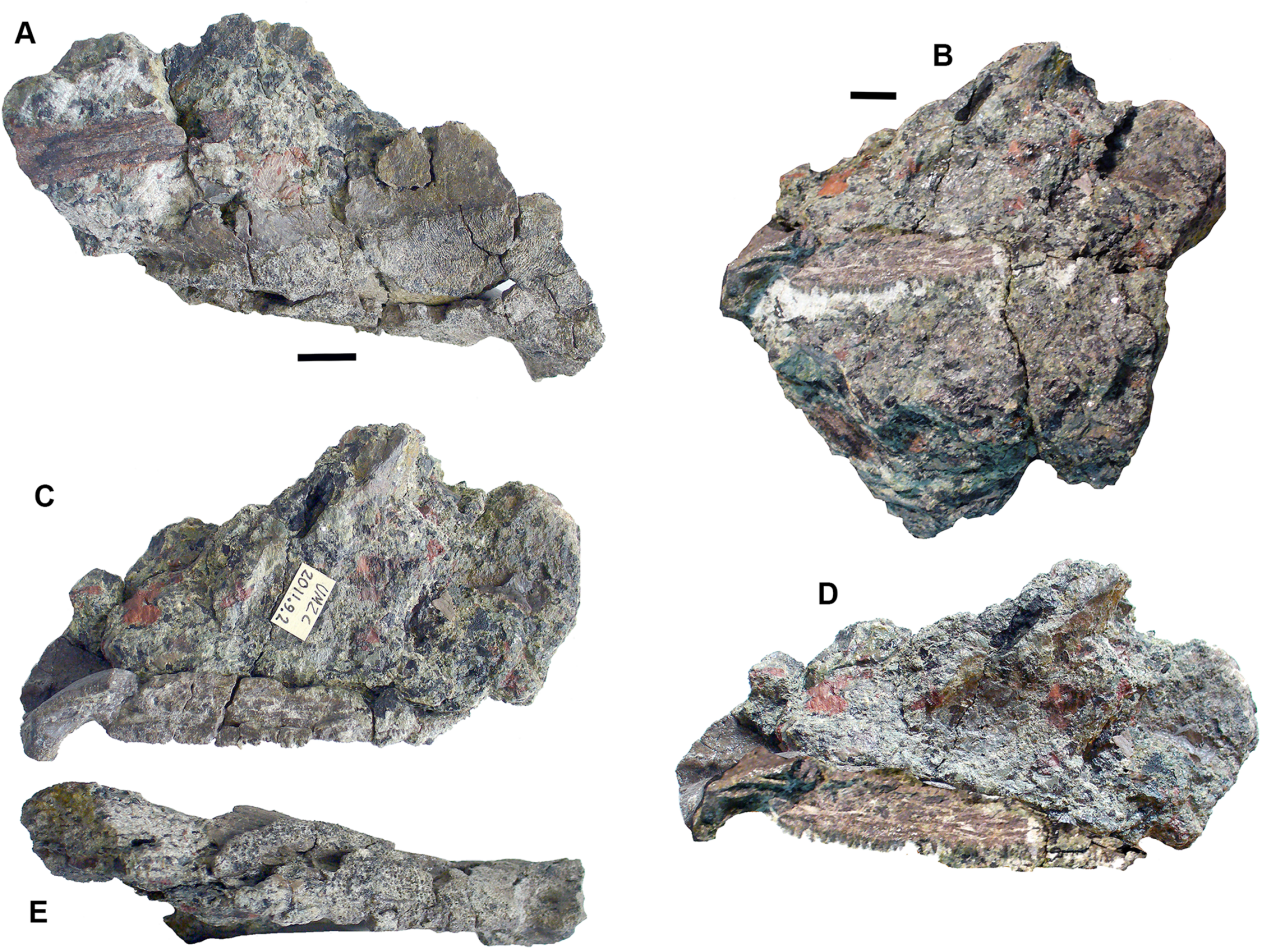
Rhizodont pterygoid 2011.9.2. (A) View of surface showing denticulated area, vermiform ornament and transition to plain surface, articular end at right. (B) View of overlap surface with teeth along the edge before preparation. (C) Same as (B) but after preparation, articular end at left. (D) Restoration of (B) plus (C). (E) View of assumed ventral surface, articular end at left. All scale bars 10 mm.

*Palate*: A partial pterygoid, 2011.9.2 (Fig. 11), is one of the few known rhizodont pterygoids, which scarcely appear in the literature. Young, Long & Ritchie (1992, figs 30 and 31) described what they considered to be the palatoquadrate and part of the lower jaw of the rhizodont *Notorhizodon* but Johanson & Ahlberg (2001) concluded that these elements probably belonged to a tristichopterid. However, 2011.9.2 bears ornament strikingly similar to that carefully illustrated in Young, Long & Ritchie’s (1992, figure 31). It consists of fine denticulations that morph gradually into vermiform grooves. There is an abrupt transition into a smooth overlap surface, exactly as depicted by Young, Long & Ritchie (1992).

A problem arises in that tristichopterids are not known to occur in the Carboniferous, having become extinct at the end of the Devonian. Our specimen is thus unlikely to be a tristichopterid. An alternative explanation is that the awkward fit between parts of the jaw and palate described by Young, Long & Ritchie (1992), and that was noted by Johanson & Ahlberg (2001), result from the parts belonging to different individuals rather than different taxa.

The bone described here is possibly much distorted. One end curves out of the plane of the denticulated surface and is smooth underneath. It appears to represent an articular surface, presumably part of the jaw joint. The surface then curves away from an edge rimmed by small teeth (unfortunately damaged during preparation and restored photographically from before and after photographs in Fig. 11D). Along the side opposite to the denticulated surface, the bone is folded downward to a ledge, presumably also an overlap surface, and the row of small teeth runs along the edge of the fold. Comparison with the Young, Long & Ritchie’s (1992) figure 31 shows that contrary to that bone, the articular end here is continuous with the denticulated ornament, whereas in the *Notorhizdon* bone, it is separated from the ornamented area by the smooth overlap surface. The relative positions of the row of small teeth and the denticulated region in 2011.9.2 is more similar to that of the palatoquadrate of *Notorhizodon*, although the entire extent of the more medial parts of the bone are missing in that specimen. There would have been some contact with the parasphenoid mesially, possibly though a quite extensive smooth overlap area, and the ledge beneath the row of small teeth would be a contact for the marginal palatal bones.

**Figure 12:**
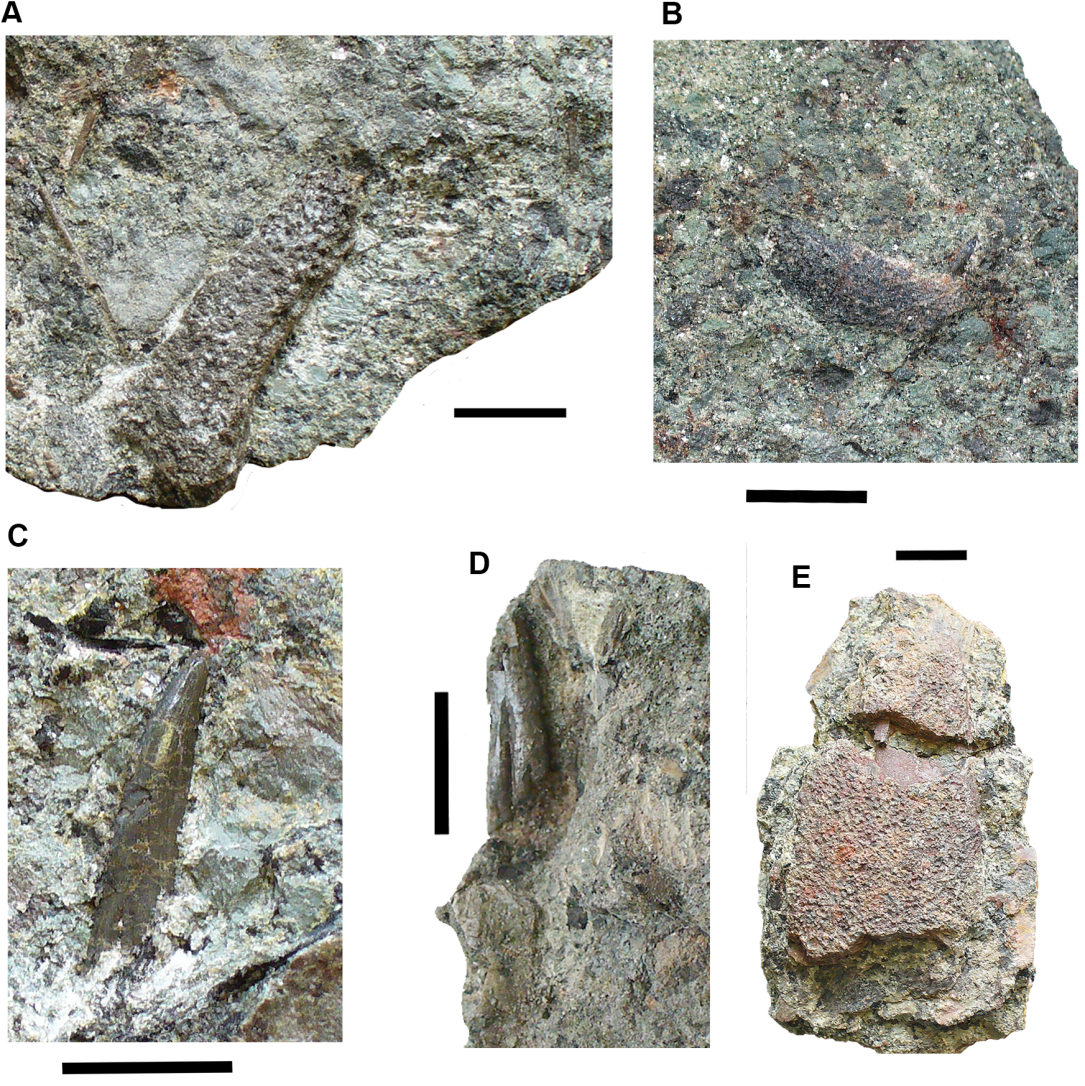
Rhizodont jaw elements and teeth. (A) Infradentary 2017.2.477. (B) Possible small dentary 2017.2.64. (C) Tooth 2017.2.72b. (D) Tooth 2017.2.99. (E) Gular plate 2017.2.64.

*Other cranial elements*: Mandibular bones include 2017.2.477 (Fig. 12A), an elongate almost rectangular bone with pustular ornamentation best identified as an infradentary; 2017.2.404 (Fig. 11B) a tooth-bearing bone, probably a small, incomplete dentary with a symphysial tusk; and 2017.2.64 (Fig. 12E), an incomplete gular plate with pustular ornamentation.

Teeth are not usually well preserved in this bed, but a few isolated examples show characteristic features. Tooth 2017.2.99 (Fig. 12D) lacks its tip but shows the folded enamel at the base. The surface is damaged, but fine almost parallel striations are visible further away from the base. Tooth 2017.2.72b (Fig. 12C) has a complete tip, which shows no recurvature like that of *Strepsodus sauroides* (Jeffery, 2006). The striations more closely resemble those of *Archichthys*, but the tooth itself is more slender than those of the holotype series. Some of the external enamel is damaged, particularly along the convex (labial) surface, so that it is difficult to say that it lacked striations as in that genus. The concave (lingual) surface shows fine parallel striations similar to those of 2017.2.99 and *Archichthys* (Jeffery, 2006).

**Figure 13:**
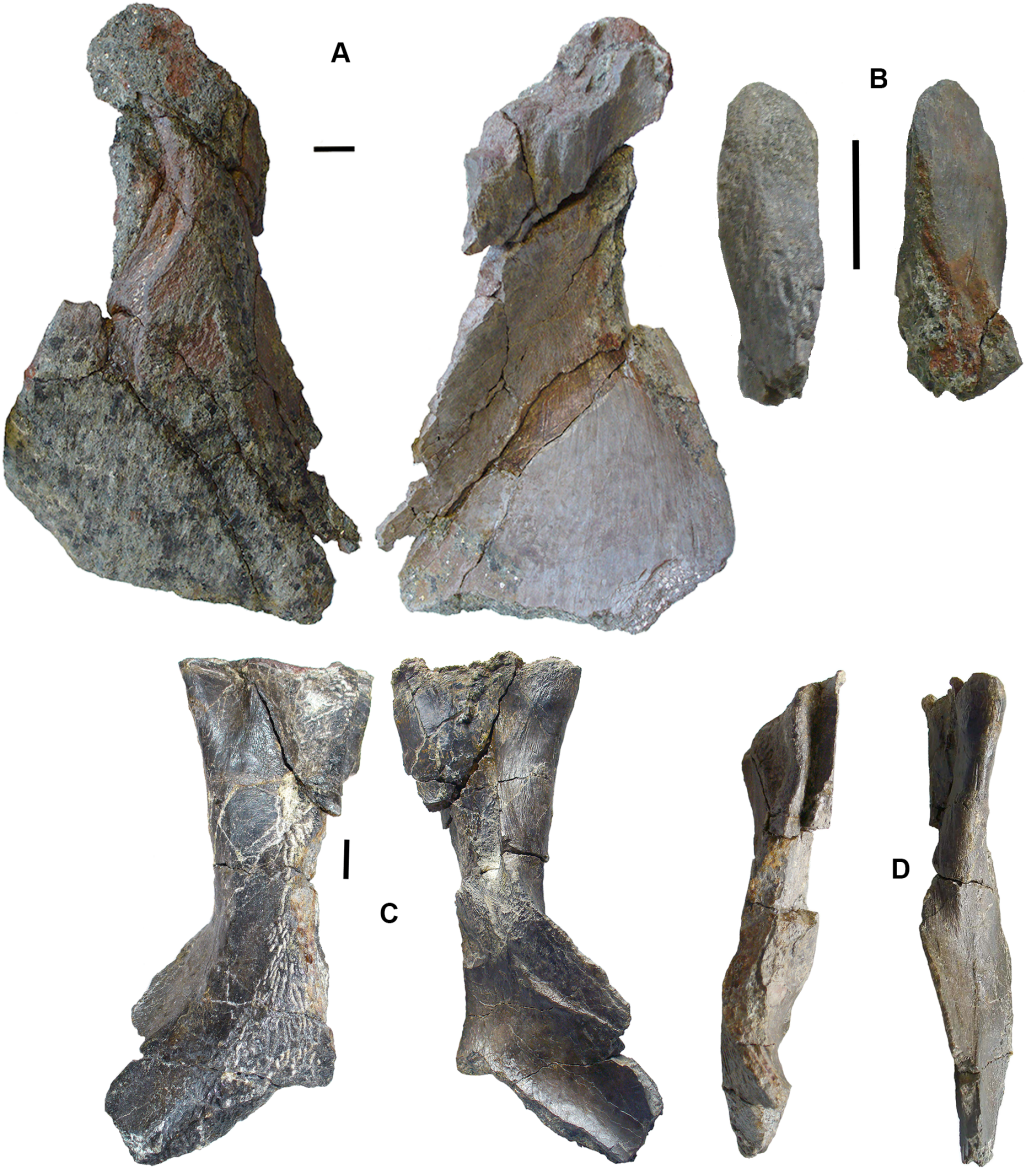
Rhizodont cleithra. (A) A very large example 2017.2.716, external view at left, internal view at right. (B) The smallest example 2018.1.4. (C) 2011.9.3. External view at left, internal view at right. (D) An eroded example 2017.2.399. All scale bars 10 mm.

*Shoulder girdle elements*: Cleithra (Fig. 13) are among the elements more commonly recovered in the collection. A large example of a left cleithrum 2011.9.3 (Fig. 13C), is incomplete dorsally and ventrally. The external surface shows coarse meandering and anastomosing ridges on the lower blade and posterior edge, but is plain elsewhere. There is a deep groove along the posterior margin and up the middle of the mesial surface of the blade. The bone conforms to those of *Strepsodus* spp. and *Strepsodus sauroides* (Andrews, 1985; Jeffery, 1999; Parker, Warren & Johanson, 2005).

An exceptionally large example, 2017.2.716 (Fig. 13A), in which the matrix has not been completely removed from the external surface, where exposed shows similar ornament to 2011.9.3. A grooved and ridged cleithrum tip (not figured) collected at the same time and close by does not fit onto the main specimen but was likely part of it. The smallest cleithrum found in this bed is 2018.1.4 (Fig. 13B). It preserves only the upper part of the blade. Two incomplete cleithra, 2017.2.399 (Fig. 13D) and 2017.2.400 (see below), show evidence of erosion by rolling during transport. They are similar to 2011.9.3 but are smaller.

**Figure 14:**
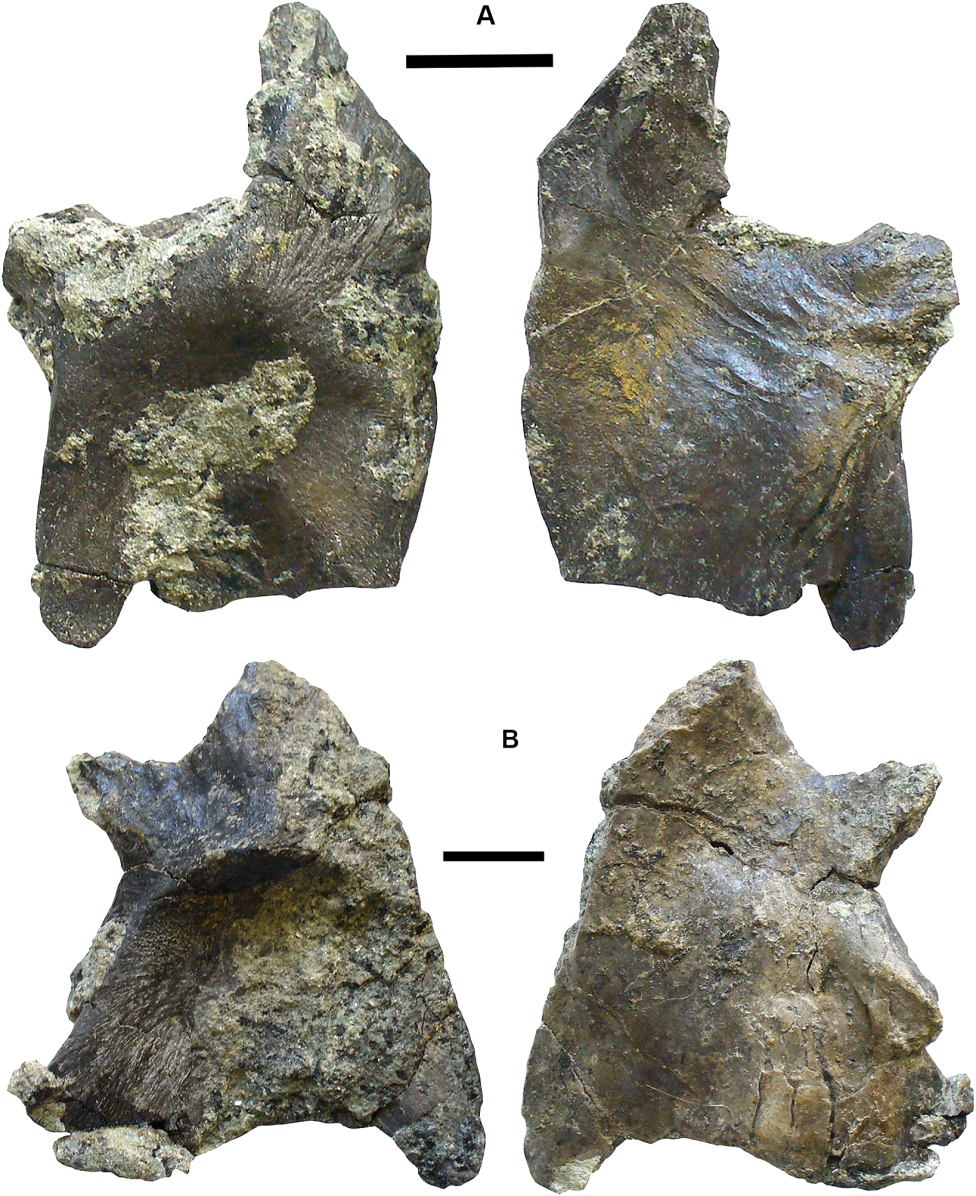
Rhizodont anocleithra. (A) 2017.2.386, probable internal view at left, external view at right. (B) 2017.2.385, probably internal view at left, external view at right. All scale bars 10 mm.

*Anocleithra*: Two specimens are identified as rhizodont anocleithra, 2017.2.385 (Fig. 14B) and 2017.2.386 (Fig. 14A), by comparison both with those illustrated by Parker, Warren & Johanson (2005) for *Strepsodus* from Ducabrook, Australia, and an unidentified rhizodont from the Dora Bone Bed, Cowdenbeath, Scotland. The two Burnmouth specimens differ in shape, although the cleithral flange in the smaller example 2017.2.386 and the dorsal flange in the larger example 2017.2.385 are both broken. Both have an anteroventral process, although that in 2017.2.386 is robust and rounded, whereas that in 2017.2.385 is pointed and more slender. Each has a clear overlap region where the cleithrum would fit, but in 2017.2.386 it is defined by a smooth rounded buttress, whereas in 2017.2.385 it is defined by a prominent ridge. Both are probably from the left side of the animal, but their cleithral flanges have distinct processes in differing positions. The lower margins of the cleithral flanges in the described examples are highly variable. It is unlikely that these two belong to the same taxon.

**Figure 15:**
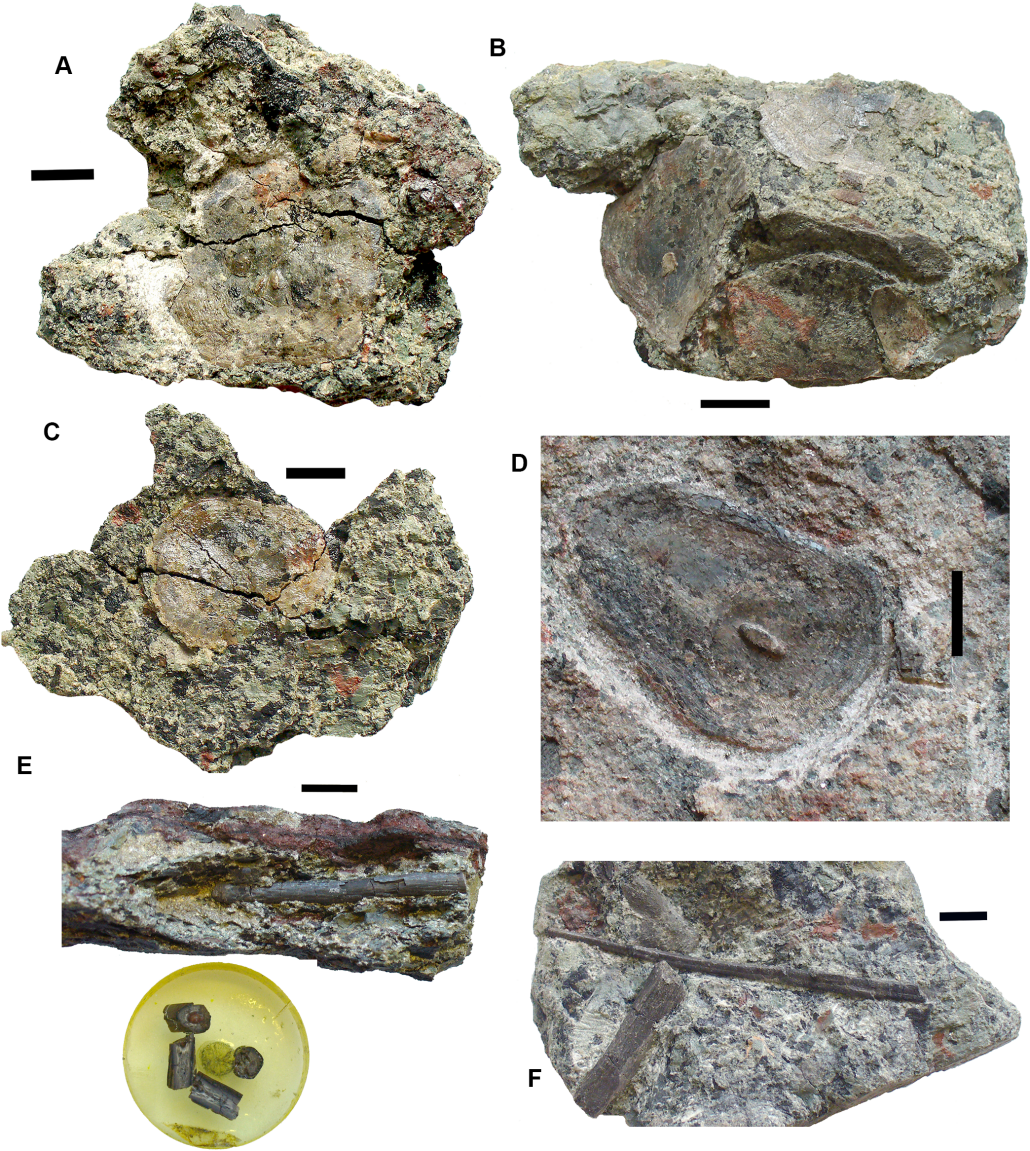
Rhizodont scales and fin spine. (A) Scale 2011.9.4. (B) Scale 2017.2.95. (C) Scale 2011.9.4. (D) Scale 2011.2.11. (E) Part of fin spine 2017.2.568, with mounted sections below. (F) Fin spine 2017.2.494b, with a partial rib of uncertain identity adjacent. All scale bars 10 mm.

*Lepidotrichia and scales*: Both large and small lepidotrichia are common in the bed. Fin spine 2017.2.494b, with about 75 mm preserved, shows a longitudinal groove, and tapers toward its distal end (Fig. 15F). Fin spine 2017.2.568 (Fig. 15E) is circular in cross section along the approximately 80 mm of its preserved length. It does not taper, but bears faint longitudinal striations. Cross sections show a thick cortex with spongy medullary bone. It was originally identified as a plant stem. The best-preserved examples of fin spines are probably attributable to rhizodonts, but may also pertain to lungfish. Rhizodont scales of many sizes are present, identified by the characteristic boss near the center of ossification (Figs. 15A–15D) (Andrews, 1985).

### Gyracanths and other chondrichthyan elements Figs. 16 and 17

**Figure 16:**
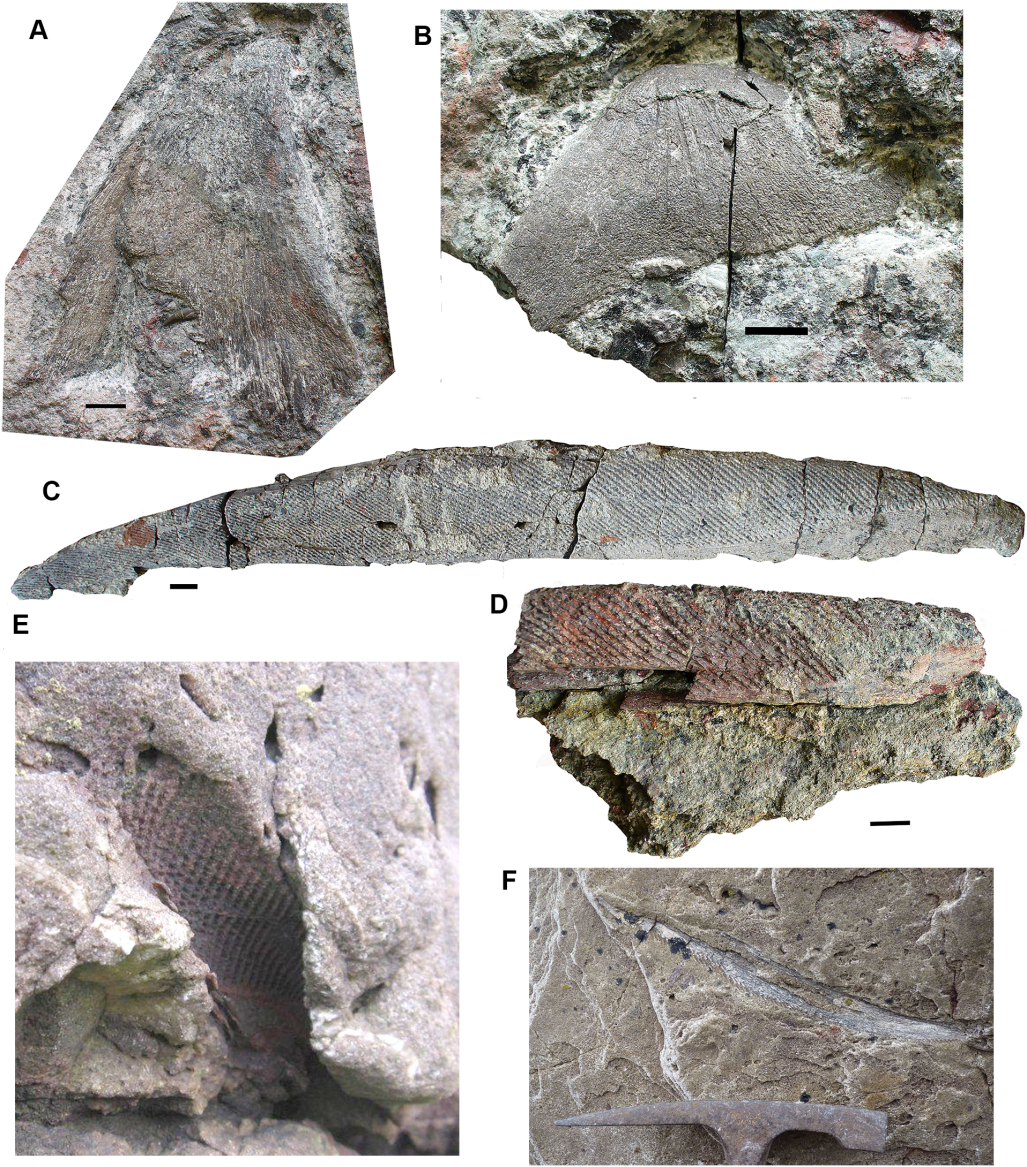
Gyracanth elements. (A) Scapulocoracoid spine 2011.9.11. (B) Procoracoid spine 2017.2.189b. (C) Pelvic spine 2017.2.182. (D) Spine 2017.2.185. (E and F) Spines preserved in situ, mostly in natural mold as seen in 2011. All scale bars 10 mm. Spine in (E) would be similar in size (when complete) to that in part (C). Hammer head for scale in (F).

Gyracanth spines are very common in this bed, although few are well preserved. Some eroded examples were to be seen in the sandstone wall above this conglomerate bed prior to the rock fall of 2018 (Figs. 16E and 16F). The most complete specimen collected is 2017.2.182, a pelvic spine measuring 40 cm. The striations are narrow, close together, and bear closely spaced tubercles. In other specimens such as 2017.2.185, an incomplete example, the ornamentation appears coarser than in 2017.2.182. There are several other examples like this. Large scapulocoracoid and procoracoid plates include 2011.9.11 and 2017.2.189b (Figs. 16A and 16B).

**Figure 17:**
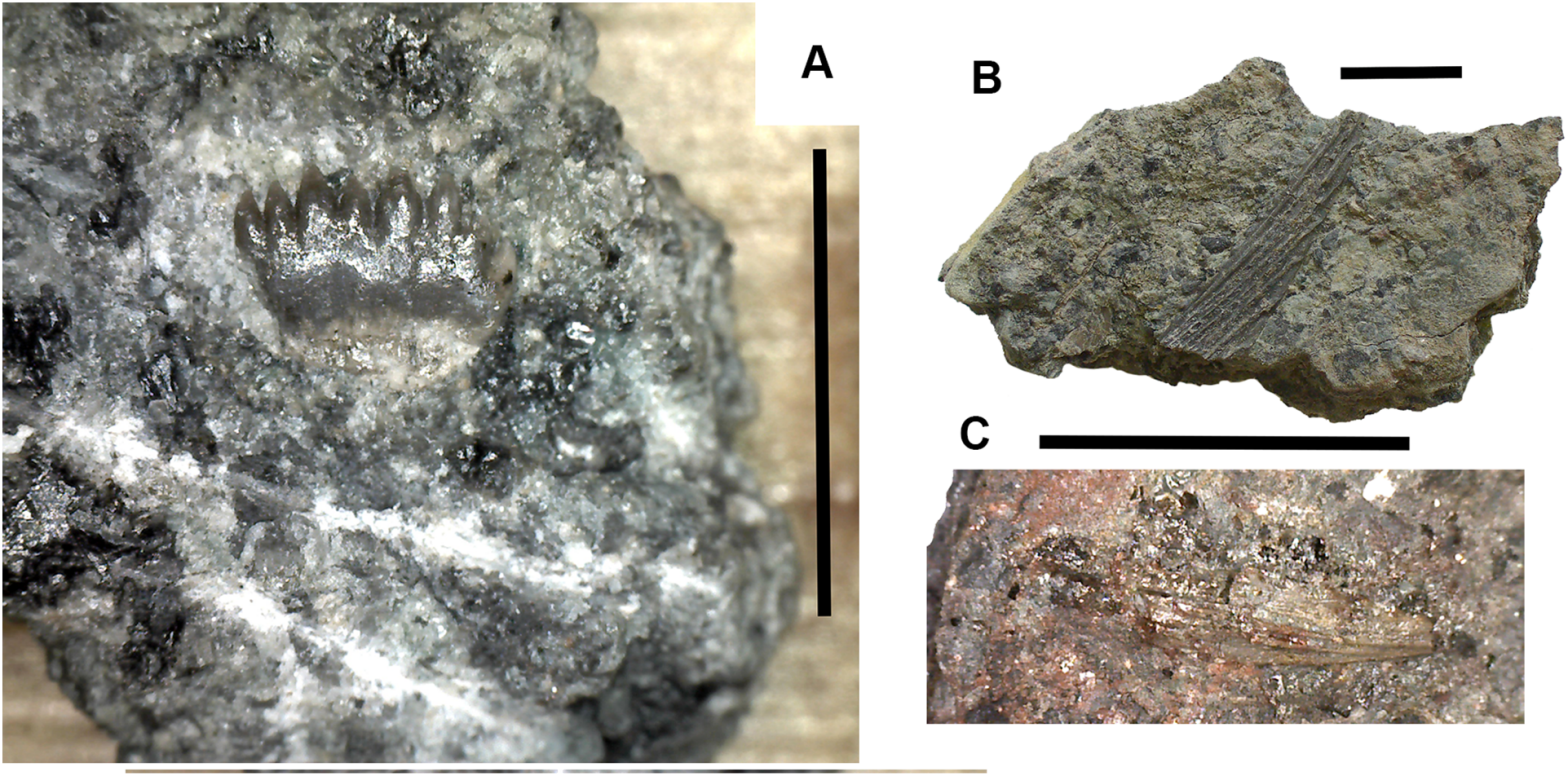
Chondrichthyan elements. (A) *Ageleodus* tooth 2017.2.282. (B) *Onychoselache*-like spine 2017.2.282. (C) Spine 2017.2.51. Scale bars for (B) and (C), 10 mm, for (A), five mm.

*Agelodus* teeth are occasionally found. The crown of *Ageleodous* tooth 2017.2.282 (Fig. 17A) originally had eight cusps, but one is missing. Cusps sit on a raised area, with the “root” depressed distally (see also the micropaleontology section for a different type of tooth).

*Onychoselache*-like spines are represented by 2017.2.397 (Fig. 17B), a small spine showing approximately longitudinal grooves and ridges, and 2017.2.51 (Fig. 17C) a smaller example.

### Actinopterygian remains Fig. 18

**Figure 18:**
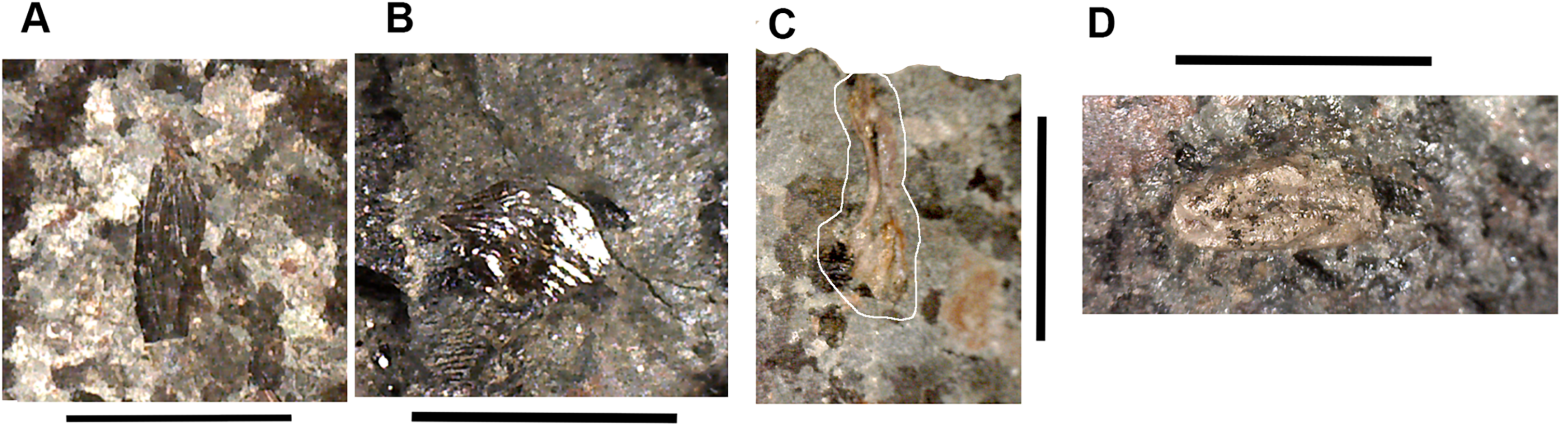
Actinopterygian elements and coprolite. (A) Scale 2017.2.200. (B) Scale 2018.1.3. (C) Possible skull or girdle bone 2018.1.1. (D) Coprolite 2017.2.58. Scale bars in (A and B) five mm, (C and D) 10 mm.

Actinopterygian scales are not infrequent in this bed (see below), but are often fragmentary. More complete examples include 2017.2.200 and 2018.1.3 (Figs. 18A and 18B). Isolated bones, though rare, may include 2018.1.1 (Fig. 18C).

### Coprolites Fig. 18

A few coprolites such as 2017.2.58 (Fig. 18D) have been found but cannot be associated with any particular taxon.

### Elements of uncertain identity Fig. 19

**Figure 19:**
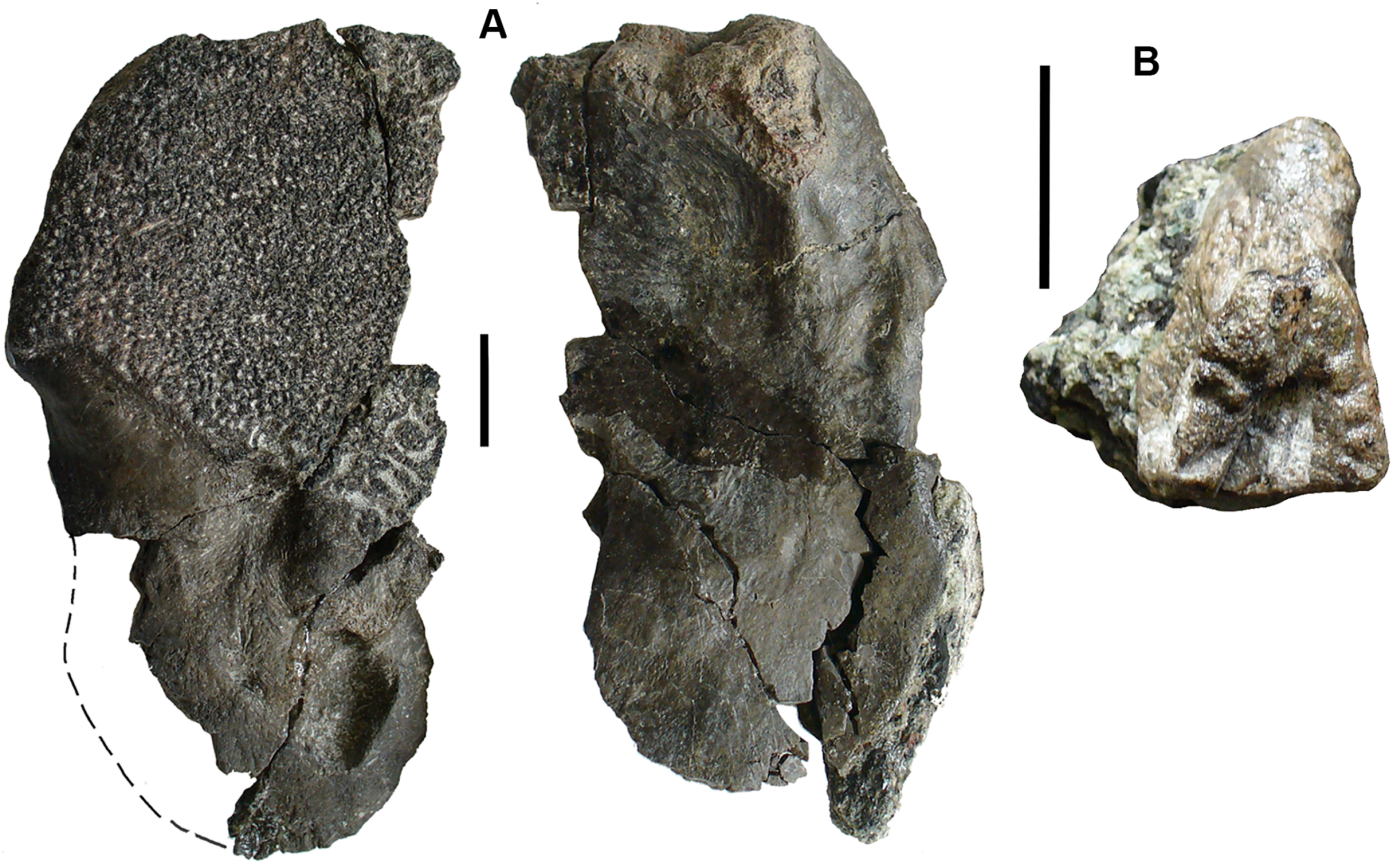
Elements of uncertain identity. (A) Large bone, possibly rhizodont 2017.2.327. (B) Unknown element 2017.2.49. All scale bars 10 mm.

A small tooth-shaped triangular element 2017.2.49 (Fig. 19A), probably flat on the unseen surface, has a rounded profile toward the tapered end, but the wider end is characterized by a raised horseshoe-shaped rim with fluting, grooves, and ridges. This bounds a depressed area. The wider end is broken, and this bone may be a small part of a larger and more complex one.

Element 2017.2.327 (Fig. 19B), appears to be from a rhizodont. It bears ornamentation similar to skull bones, including some vermiform grooves in a small area. One margin, the left side in the figure, is strongly curved downward. The other is broken. The ornamented area terminates in a distinct margin. Extending from this is an unornamented region with a smooth convex outline. It is marked on this surface by a deeply depressed area toward the side opposite the curved margin. On the underside, the whole surface is smooth, but there is a buttress beneath the curved margin that bounds a depressed and grooved area. There is no obvious distinction on the internal surface between where the ornamented segment of the external surface of the bone ends and the smooth area begins.

Suggestions for the identity of this bone include part of the shoulder girdle, a quadratojugal with attached quadrate, a surangular with attached articular, and a pterygoid with attached epipterygoid. We welcome other suggestions.

### Abraded elements and plant macrofossils Fig. 20

**Figure 20:**
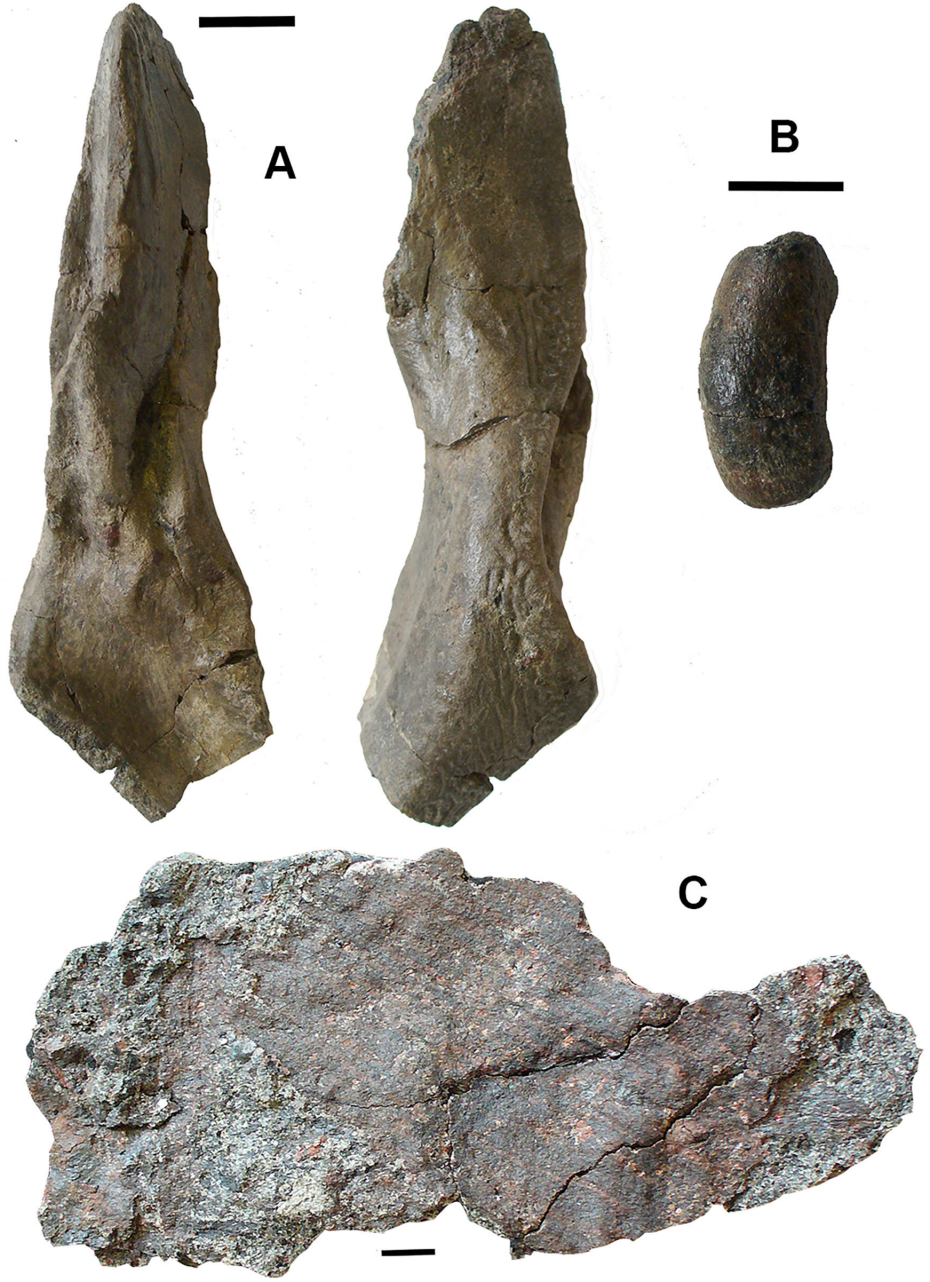
Abraded elements and plant remains. (A) Rhizodont cleithrum 2017.2.400. (B) Rolled bone 2017.2.214. (C) Plant stem, probably lycopod 2017.2.293. All scale bars 10 mm.

Rolled bones such as 2017.2.214 of uncertain identity (Fig. 20B), appears smooth and featureless. It is convex in profile and slightly C-shaped. It may be part of a rib that has been rolled during transport. Other rolled and eroded bones include the two rhizodont cleithra listed above (Figs. 13D and 20A).

Plant macrofossils are common with some large elements represented (Fig. 20C), suggesting that large plants existed during that part of the Tournaisian. However, they are usually poorly preserved such that identification is not usually possible. Some appear to be lycopsid. They are often coated with iron oxide, as are some of the bones (see below).

### Microfossil assemblage Figs. 21 and 22

**Figure 21:**
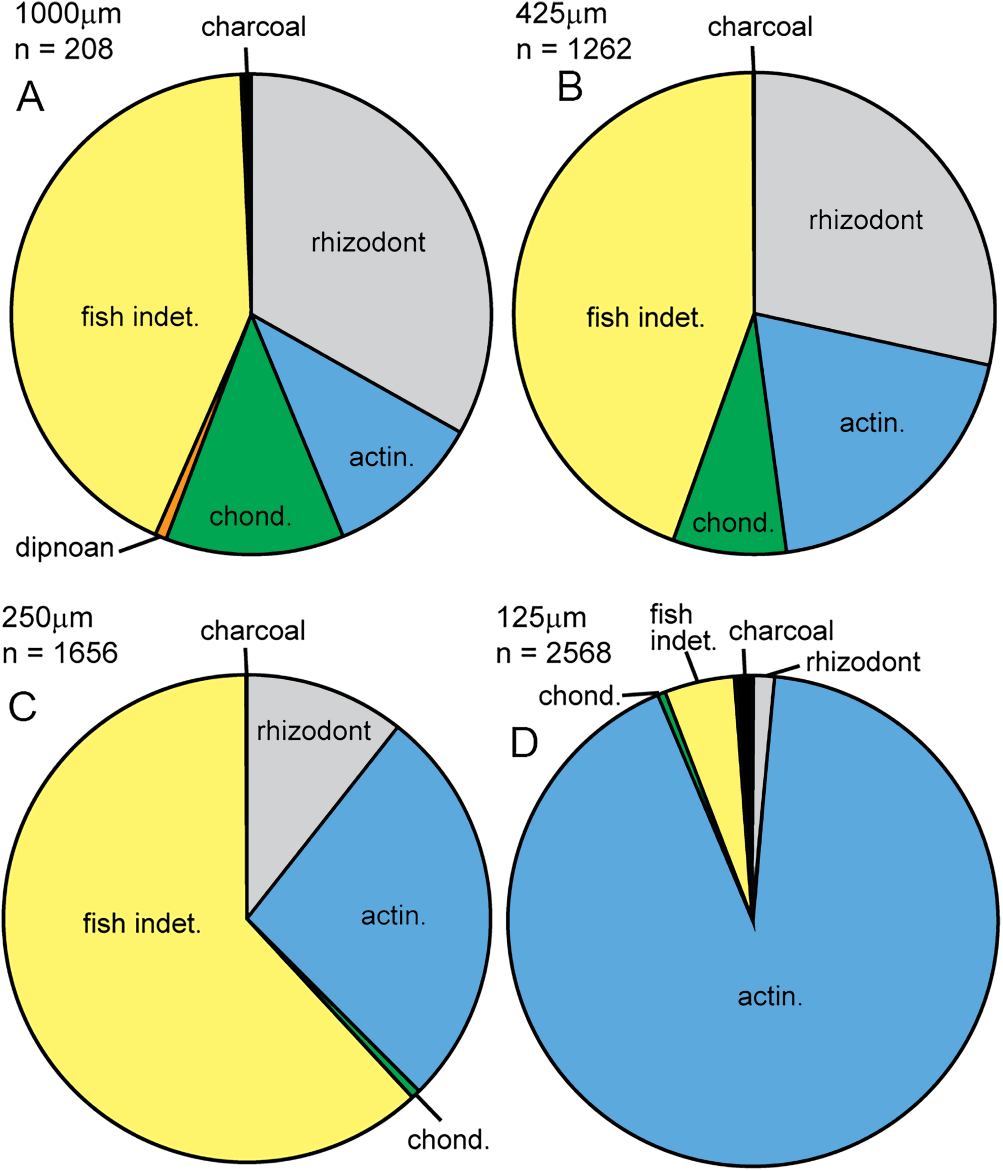
Micropaleo pie chart. Microfossil assemblage. Percentage plots of microfossil counts for the four size fractions analyzed: (A) 1,000 μm fraction. (B) 425 μm fraction. (C) 250 μm fraction. (D) 125 μm fraction. The full data table of counts for all size fractions is presented in Supplementary Information 1. Abbreviations: actin., actinopterygian; chond., chondrichthyan; indet., indeterminate.

In this study, we compared the microfossil assemblage to the macrofossil assemblage in Bed 383, confining the identity of specimens to major groups. The micropaleontological assemblage (SI1) is almost entirely composed of vertebrate material (5,660 out of 5,694 specimens), with some charcoal fragments (34 specimens). Microfossils are generally well preserved, although some abrasion, wear surfaces and cracks are observed. For vertebrates, 67.9% of specimens recovered have been identified. Rhizodont and actinopterygian material dominates the assemblage, with lesser occurrences of chondrichthyan and dipnoan microfossils (Fig. 21). Charcoal is identified by its black color, brittle texture, fibrous external structure and hollow internal structure of preserved cellular tissue (see below and Scott, 2010).

**Figure 22:**
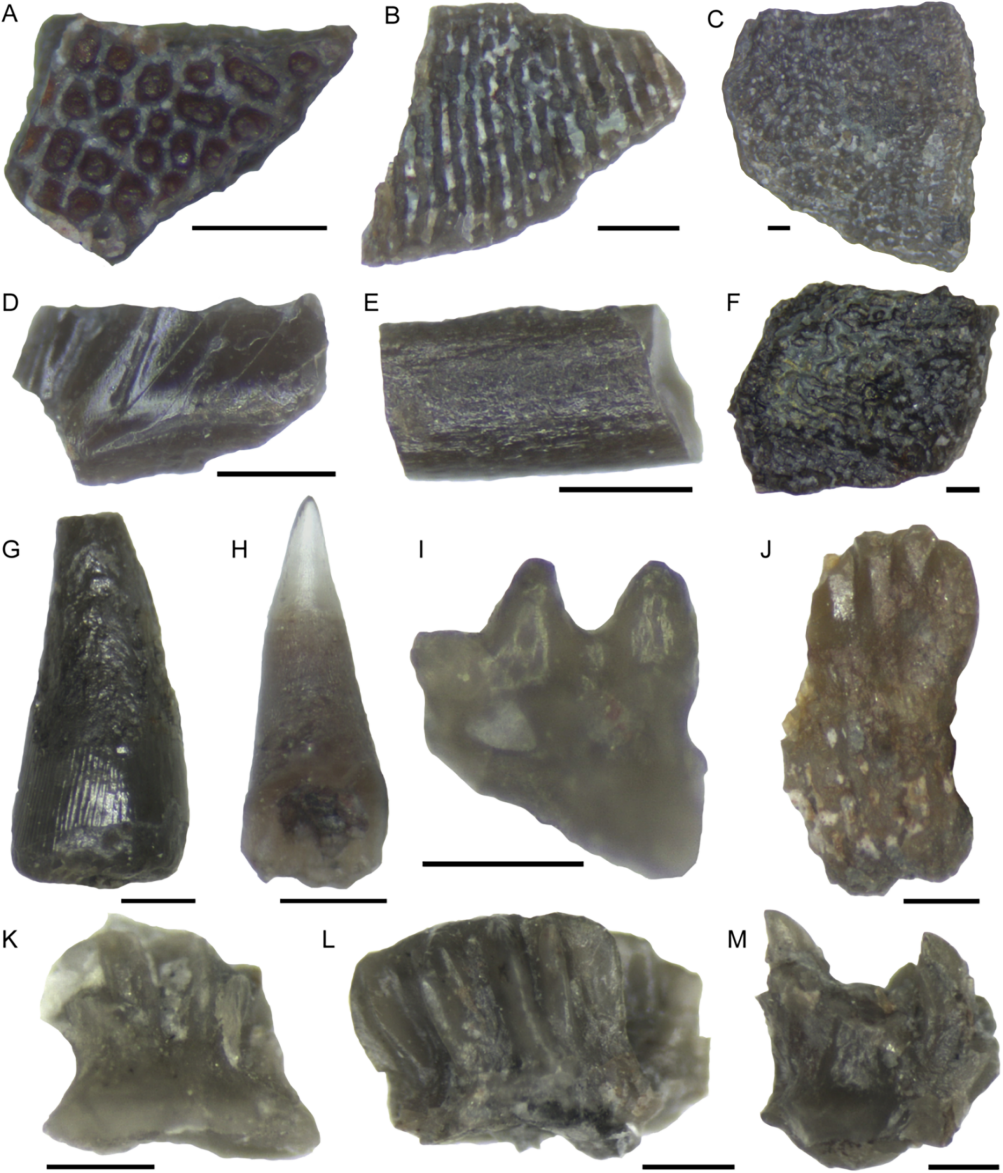
Selected microfossil specimens. (A) Rhizodont scale with tuberculate internal structure and red staining. (B) Rhizodont scale with ridge and groove internal structure. (C) Rhizodont dermal bone, view of the exterior surface with irregular tuberculate ornamentation. (D) Actinopterygian scale fragment, exterior surface with transverse ridges and grooves. (E) Actinopterygian lepidotrichia fin bone fragment. (F) Actinopterygian dermal bone, exterior surface with irregular tuberculate ornament and gamine coating. (G) Rhizodont tooth with striations at the base, lateral view. (H) Actinopterygian tooth, with transparent cap, lateral view. (I) pharyngeal actinopterygian tooth, double row, lateral view. (J) *Ageleodus* tooth with broken tooth cusps, lateral view. (K) Elasmobranch scale with a concave base and spinose top, lateral view. (L) Hybodont scale with spines that are joined together into a star shape, lateral view. (M) ?Ctenacanth scale with a top of curved spines. Scale bars 250 μm.

Rhizodont scales have an exterior surface which is cream colored, shiny and has a fibrous structure. When broken, the interior layers have a range of characteristic structural elements including sheets of either tubercules and pits or grooves and ridges that interlock together (Figs. 22A and 22B). These structures were identified by the examination of broken rhizodont scale macrofossils from this bed. A total of 13.9% of the rhizodont scales have a red staining on the interior surfaces, likely due to the presence of iron oxide. Other rhizodont elements include rhizodont dermal bone (Fig. 22C) with a distinctive ornament of tubercules or ridges (cf. Jeffery, 2012) and three indeterminate rhizodont teeth (Fig. 22G), which have a shape similar to that of *Strepsodus*, but an ornament of well-defined striae similar to that of *Archichthys* (Jeffery, 2006).

Actinopterygian scales have a rhombic shape with a smooth interior surface with keel, and a shiny, sculpted exterior surface (Fig. 22D) with transverse ridges or grooves (cf. Carpenter et al., 2014). Actinopterygian dermal bones and lepidotrichia (Fig. 22E) are also identified. Some of the dermal bone is similar to that of rhizodonts, with pustulate ornament on one side, but the fragments are small and they have a shiny, ganoine surface texture (Fig. 22F). The lepidotrichia are most common in the 250 μm size fraction, so are more likely to be actinopterygian than rhizodont, although there are no physical characteristics to distinguish them other than size. They have a range of surface textures, from smooth to longitudinal striations or ridges. The interior medullary bone of the fine spines is infilled with calcite in some specimens. Actinopterygian teeth are present in all apart from the 1,000 μm size fraction and are distinguished by their transparent apical cap (Fig. 22H) and shaft with cross-hatched surface ornamentation (Carpenter et al., 2011). Pharyngeal actinopterygian teeth are unornamented, curved, blunt at the tip, and occur in rows (Fig. 22I). In total 30 main teeth and 92 pharyngeal teeth are recorded.

Chondrichthyan teeth comprise three *Ageleodus* teeth with five to six tooth cusps (Fig. 22J) and one xenacanth (indeterminate) tooth (partly broken). Chondrichthyan material is dominated by denticles (140 specimens), which are common in the 1,000 and 425 μm fractions and are mostly present as fragments in the 250 and 125 μm fractions. Three types of scales have been identified; elasmobranch (*n* = 43), hybodont (*n* = 28) and ?ctenacanth (*n* = 5). Indeterminate elasmobranch scales are identified by a flat or concave base and a top of curved spines (Fig. 22K), which in dorsal view form clusters of irregular height, or individual spines (Burrow, Long & Trinajstic, 2009; Carpenter et al., 2011). Hybodont scales have a concave base, spinose top and distinctive grouping of spines (Fig. 22L) which forms a single flat star-shape, or multiple star-shaped clusters in dorsal view (Garvey & Turner, 2006; Yazdi & Turner, 2000). Scales which can be putatively assigned to a ctenacanth origin have a flat base, spinose top and similar appearance to contemporaneous specimens described by Ivanov (1996) and Yazdi & Turner (2000). The spines are strongly curved, numerous and are of irregular height (Fig. 22M).

Tetrapod microfossils have not been recorded from this bed and only one dipnoan element is identified (a toothplate fragment). This may result from problems in identification, given they are more common as macrofossils. Further direct comparative work on broken dipnoan and tetrapod macrofossils is necessary to determine their microfossil characteristics.

### Charcoal description and identification Figs. 23–26

**Figure 23:**
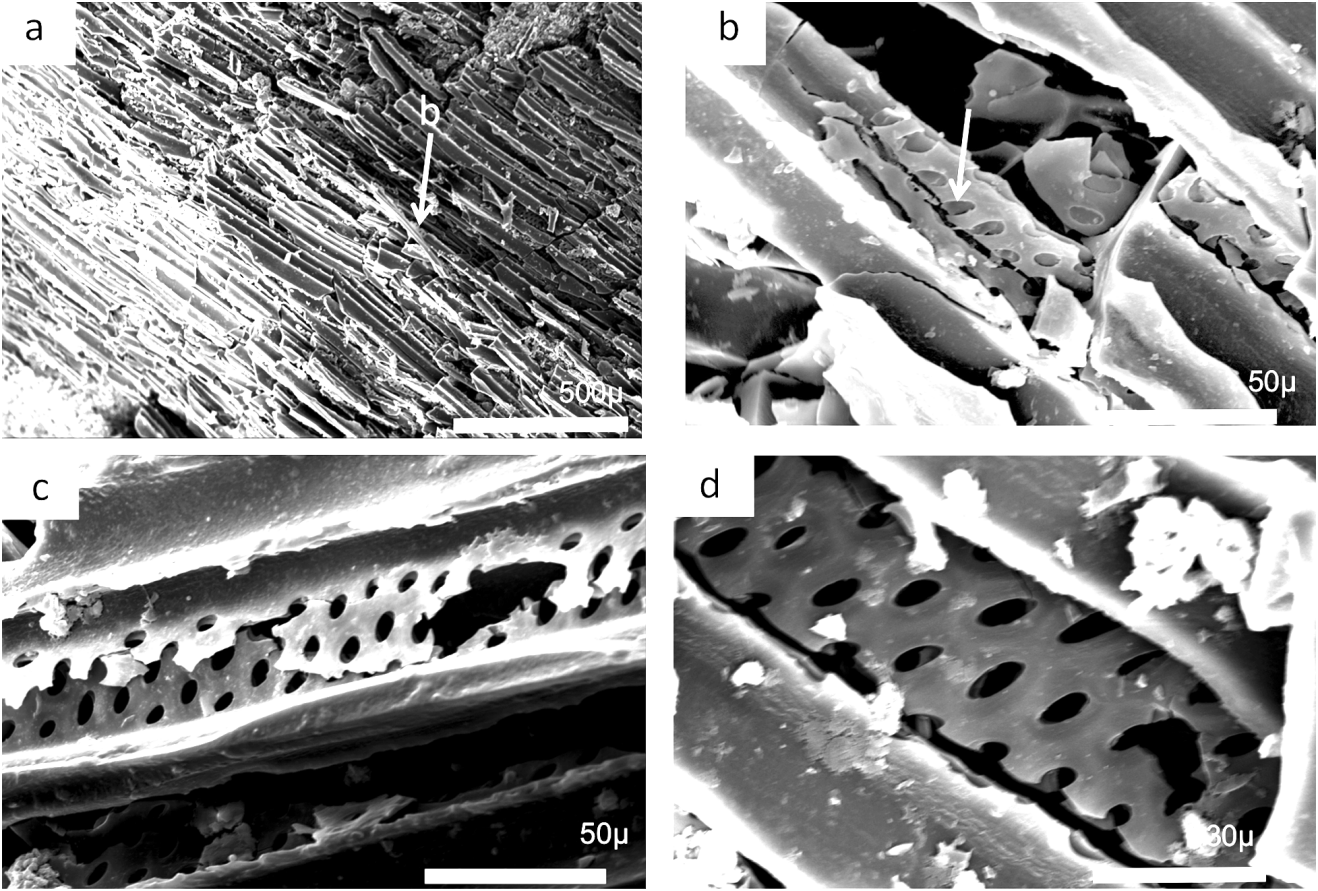
Scanning electron micrographs of charcoal fragment from Bed 383. (A) Longitudinal section showing pitting in the cell walls. (B) Detail of (A) showing rows of circular pits. (C) Detail of (A) showing three rows of circular pits. (D) Detail of (A) showing three rows of circular pits.

The charcoal fragments in Bed 383 range in size from a few millimeters to a centimeter in maximum dimensions (Fig. 23). All are woody, comprising both secondary xylem and ray cell tissue. All specimens show evidence of pitting in the tracheid cell walls that are often circular pits arranged in single or in several rows (Figs. 23B and 23D). The cells also show evidence of homogenization (Fig. 23B) (Scott, 2010). It is often considered that secondary xylem (wood), that is, dense or pycnoxylic is the most common feature of the arborescent pteridosperms (Galtier & Meyer-Berthaud, 2006) (Table 1). The tracheids are often less than 50 μm wide and have short uniseriate rays, but this is not easily visible. There is insufficient detail in the specimens found so far in Bed 383 to provide definitive identifications.

**Table 1:** Characters of secondary wood of Tournaisian Pteridosperms.

Genus	Pycnoxylic	Manoxylic	Rays (wide)	Tracheid pitting	Rays height
*Dadoxylon*	×		3 cells	Multi-3 seriate/circular	
*Calamopitys*		×	1–11	Up to 7 seriate hexagonal	to 200 cells
*Pitus*	×		1–8 to 15	3–4 seriate hexagonal to circular	to 60/to 100
*Eristophyton*	×		1–2 to 7	3–4 seriate hexagonal to circular	to 10–25
*Bilignea*	×		1–2	1–2 seriate circular	to 10
*Endoxylon*	×		1–2	1–2 seriate circular	to 10

**Note:**

Data from Galtier & Meyer-Berthaud (2006) and references therein.

**Figure 24:**
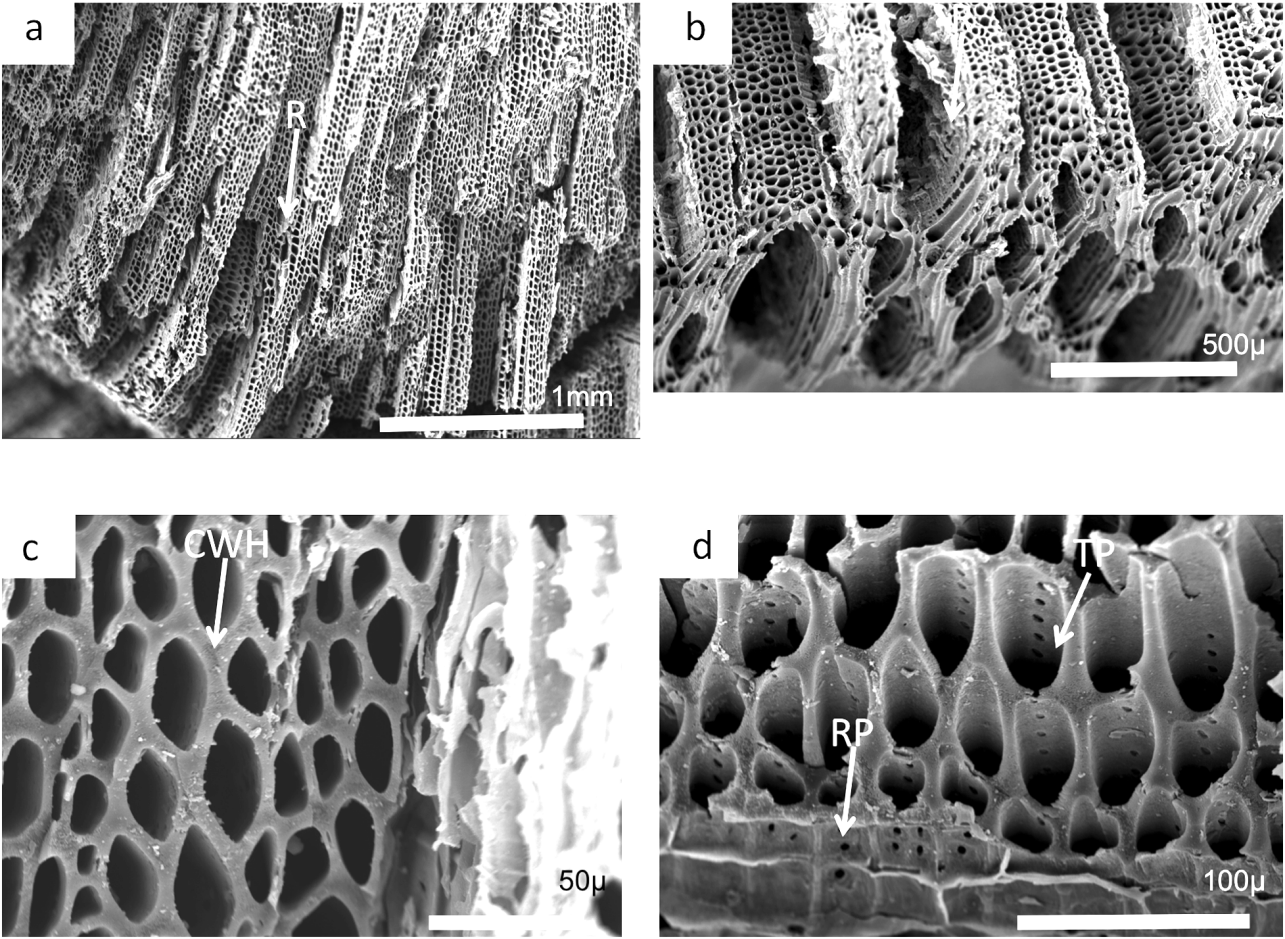
Scanning electron micrographs of large charcoal fragment from Bed 362. (A) Low power image showing rows of tracheids separated by ray tissue. (B) Detail of (A) showing the position of short rays. (C) Detail of tracheids showing cell wall homogenization. (D) Detail of tracheids showing single rows of circular pits and ray cells with pits.

**Figure 25:**
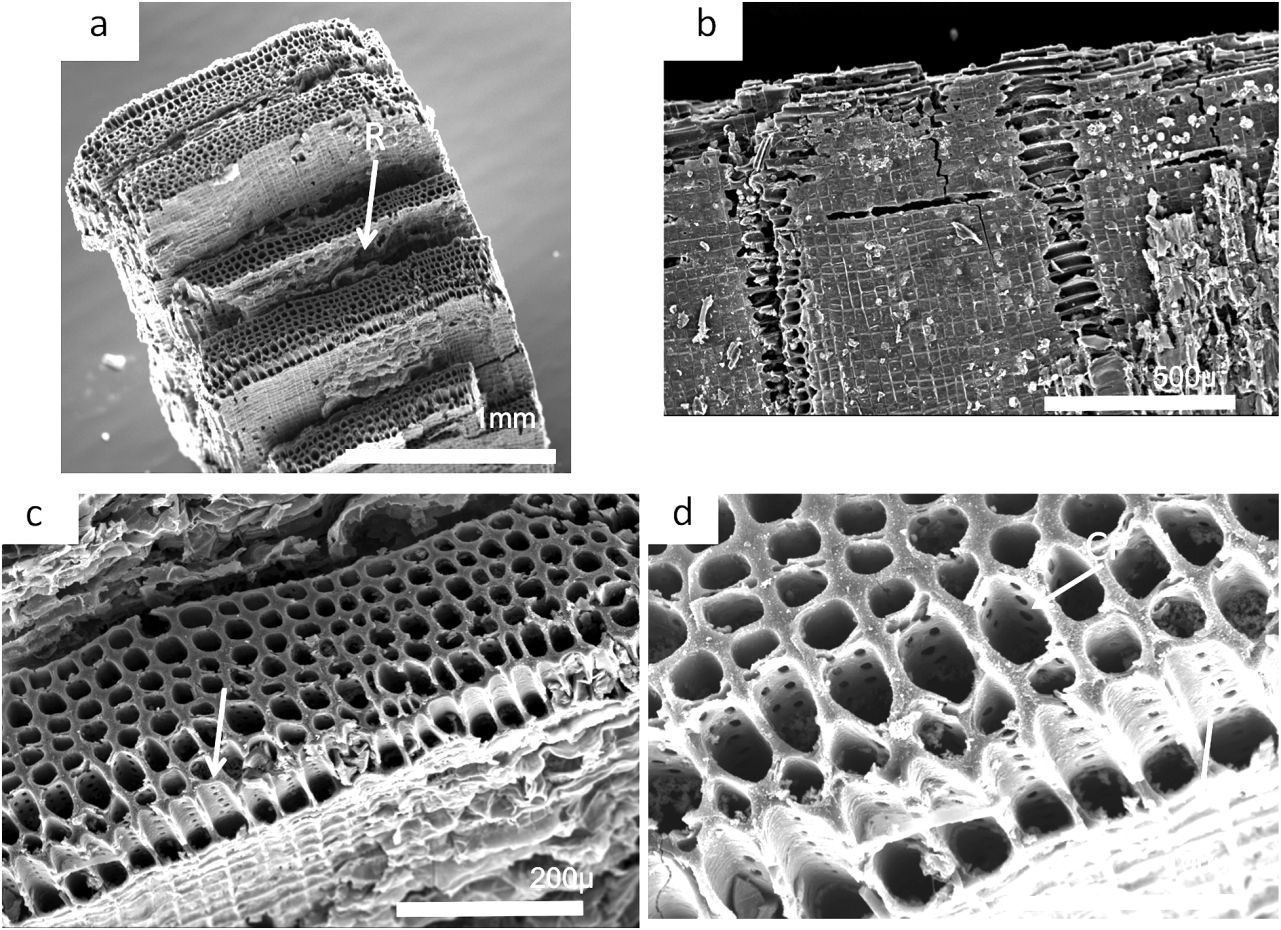
Burnmouth charcoal Bed 362.2. Scanning electron micrographs of charcoal fragment 1 from Bed 362 (UMZC 2018.4.13). (A) Wood fragment showing the position of ray tissue (R). (B) Detail of (A) showing ray tissue. (C) Detail of (A) showing pitting in tracheids (arrow) (D) Detail of (A) showing rows of circular pits (CP), homogenized cell walls and ray tissue (R).

**Figure 26:**
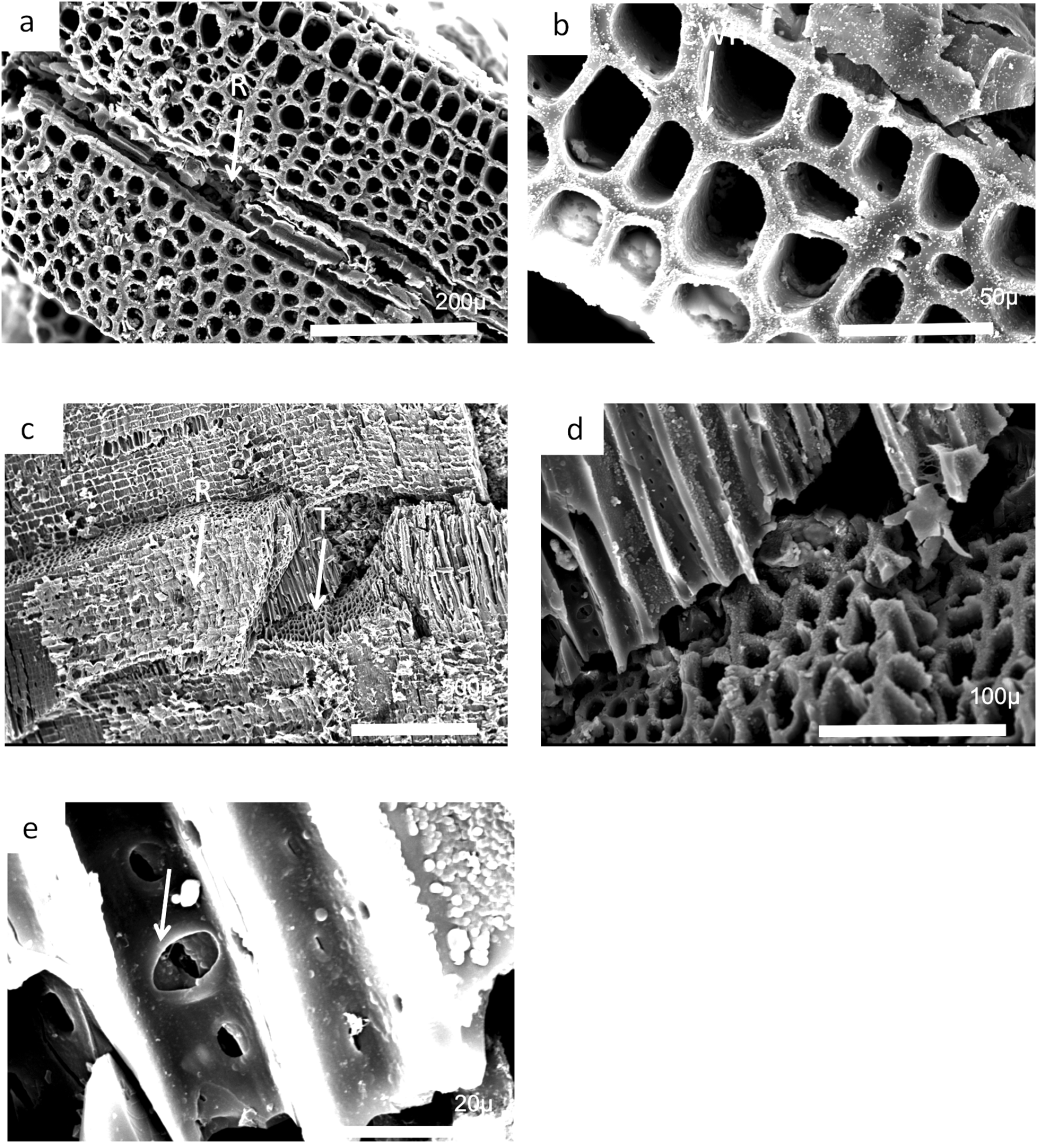
Burnmouth charcoal Bed 362.3. Scanning electron micrographs of charcoal fragment 2 from Bed 362 (UMZC 2018.4.13). (A) Specimen showing rows of tracheids separated by ray cells (R). (B) Detail of tracheids showing cell wall homogenization (CWH). (C) Specimen showing both tracheids (T) and ray tissue (R). (D) Detail of (A) showing tracheids. (E) Circular bordered pit (arrow)n tracheid.

Charcoal fragments from Bed 362 (2018.4.13) are expected to preserve similar taxa to those of Bed 383, but they have a greater range of size, many specimens ranging from 10 to 20 mm in maximum length (Figs. 24 and 25). In addition, because of their larger size they show a wider range of characteristics including relatively small xylem cell sizes (Figs. 24B, 24D, 25C and 25D) but large numbers of wide rays (Fig. 24B). However, the specimens in Figs. 25A, 25B and 26C expose wide surfaces of longitudinal radial sections split at the level of the “soft” ray tissue, demonstrating that rays were very high (i.e., tens of cells high). They may exhibit several types of wall pitting in the xylem as well as in the ray cells (Figs. 25B and 25D). The pitting may comprise several rows of pits (Fig. 25D) and occasionally bordered pits (Fig. 26C). In one specimen (Fig. 24) there appears to be the presence of leaf traces (Fig. 24B) and also regular rays separating a few rows of tracheids (Figs. 24A and 24B). Cell wall homogenization is also seen (Fig. 26B).

Most of the charcoal appears to have come from small woody axes and possible larger wood from a range of arborescent pteridosperms (Galtier & Meyer-Berthaud, 2006). Such pteridosperms are widely reported throughout the Mississippian of Scotland (Scott, Galtier & Clayton, 1984; Scott & Galtier, 1988, 1996; Galtier & Meyer-Berthaud, 2006). Most common taxa that may be represented at Burnmouth include the large tree *Pitus*, that is, also common in the Borders (Long, 1979) and the smaller *Eristophyton* (Scott & Galtier, 1996; Galtier & Scott, 1990). Other taxa reported from Tournaisian rocks in the Midland Valley (Galtier & Meyer-Berthaud, 2006) include *Bilignea*, *Endoxylon*, and *Buteoxylon*, although the size of the charcoalified specimens do not allow the preservation of the combination of characters that will allow their certain identification.

Some of the specimens are of pycnoxylic wood, that is, compact with little parenchyma between the rows of tracheids (Fig. 26A). *Dadoxylon* wood may also be a candidate as they have uni- to biseriate rays. *Calamopitys*, in contrast, has been characterized as having manoxylic wood in the stems (i.e, abundant parenchyma cells between rows of tracheids) and a thick cortex with a range of cell sizes. The genus is more shrub-like with stems that are only two to three centimeters in diameter. A related form *Stenomyelon,* however, has been reported from Scotland (Scott & Galtier, 1988). All the specimens we have seen have a nonmanoxylic wood and cannot be referred to calamopityans. In *Pitus* (*Pitys*) the tracheids of the secondary wood typically have pits arranged in multiseriate rows (Gordon, 1935) and the trunks of this plant can be very large and may provide significant quantities of wood that are available to produce charcoal. Characters differ, however, between species of this genus (Galtier & Meyer-Berthaud, 2006).

In many cases small fragments of wood charcoal are difficult to identify as the tracheid dimensions and pitting types occur in many different taxa. What is necessary are the ray size measurements and ray organization (number of cells wide and number of cells high) and this is only possible if, in addition to a good transverse section, radial and tangential longitudinal sections are available. It is possible from the limited information available that the specimen in Fig. 24 has broad rays, but since there is no evidence that they are very high, they may be comparable to *Eristophyton waltonii* (cf. Galtier & Meyer-Berthaud, 2006, fig. 5E). Specimens in Figs. 25 and 26 (possibly 26 but without ray data) have very high rays but no evidence they were broad (i.e., more than three to four cells wide) and therefore may be comparable to *Pitus antiqua* (Galtier & Meyer-Berthaud, 2006, fig. 5H).

## Discussion

### Macrofossil assemblage

The partial *Crassigyrinus*-like lower jaw, discovered in 2010 (UMZC 2011.9.1), from Bed 383, is about 20 Myr older than *C. scoticus*, and although attribution to the same genus is not unreasonable, it is unlikely to represent the same species (Clack, Porro & Bennett, 2018). Also found in 2010 were a partial parasphenoid and a basal process, not identified as *Crassigyrinus*-like at the time. These braincase elements indicate an animal at least 50% larger than *C. scoticus*.

Earlier-collected material includes central elements: one complete centrum, one half of a centrum, and one fragment. Phalanges or metapodials of a large tetrapod are also preserved, though their attribution to *Crassigyrinus* is less secure. Subsequent discoveries include ribs and body scales. Found in close proximity, probably in less than one square cubic meter of the 15–20 cm thick matrix, it is possible that many of these elements belonged to the same individual. The fact that few other elements have been found more recently suggests that most of the animal’s remains had been eroded away before collecting began.

Smaller elements among the macrofossils include a jugal (Clack et al., 2016, fig. 6), ribs with uncinate processes and an ulna. These much smaller and moderate sized bones show the presence either of small-bodied individuals or juveniles. Also included are delicate ribs, limb bones, and small phalanges. Their shapes are quite different from those in *C. scoticus* and furthermore, probably do not belong to a juvenile of the same taxon as the lower jaw.

As with the tetrapods at this bed, lungfish elements indicate a wide range of sizes of individuals, from tiny juveniles or small-bodied taxa up to large adults. At least two very large individuals are represented by opercula within this relatively small accumulation. To our knowledge, these represent the largest lungfishes found in the Carboniferous. A rough guide to the length of the fish can be obtained by a ratio of 15:1 from the diameter of the operculum (T.R. Smithson, 2017, personal observation). By this measure, the large opercula belonged to fish up to three meters in length. Those from the later Carboniferous found so far are no more than about half the size as judged by this ratio. This finding, as with some of the tetrapod, rhizodont, and gyracanth elements, is in contrast to the trends in vertebrate size documented by Sallan & Gallimberti (2015), who suggested a reduction in maximum sizes during the Tournaisian, followed by very slow recovery of larger forms later in the Carboniferous.

By contrast, no large tooth plates have been found, but several very small ones are present, including probable representatives of two species of *Ballagadus* (Smithson, Richards & Clack, 2015). Skull bones with varying surface ornamentation or none suggest that more than one taxon is present in the assemblage. Large elements with ornamented external surfaces and deeply interdigitated sutural surfaces contrast with small often oval or hexagonal elements with smooth or pitted surfaces and no obvious sutural overlap areas. Whether these differences could be accounted for by ontogeny is not clear.

Rhizodonts are represented mainly by bones from large or medium-sized individuals, although small scales, a small cleithrum and a small postparietal have been recovered. Although many specimens resemble bones of *Strepsodus* it is possible that two taxa of rhizodont were represented in Bed 383. The very large cleithrum suggests a fish of a size comparable with those of the large lungfish, again in contrast to Sallan & Gallimberti (2015).

There is some question of the taxonomic status of the rhizodonts from Burnmouth. Otoo (2015) and Otoo et al. (2018) suggest that two taxa might be present in a somewhat older assemblage from Burnmouth, possibly *Strepsodus* and *Archichthys*. This requires further investigation, but the anocleithra and the postparietals (Figs. 1A, 1B, 1E, 14A and 14B) do suggest the presence of two taxa in Bed 383. An isolated humerus from a lower bed (Bed 340.5 see also Otoo et al., 2018) under study by JAC and coauthors is quite different from those figured for *Strepsodus*.

Gyracanth spines are among the most common elements in this bed, though they are difficult to collect and prepare and are often broken. Some elements are exceptionally large, in keeping with the large elements of tetrapods, lungfishes, and rhizodonts. Similar spines, though not as long as 2017.2.182 were found in the base of the overlying sandstone (probably those noted in McAdam, Clarkson & Stone (1992), although they had been subject to erosion on the surface of the exposed wall. They were lost in the rock fall.

Rare chondrichthyan remains suggest their presence in the region at the time, and are better represented in the microfossil assemblage (see below).

One of the issues encountered in this investigation is the difficulty of identifying isolated elements. This is particularly the case with possible rhizodont elements that are known from very few other taxa. Where possible we therefore illustrate here both internal and external surfaces of individual bones. Internal surfaces of skull bones are often not accessible except by micro-CT scanning, so that it is important to illustrate both where circumstances allow. Our rhizodont skull bones also show unusual surface features of the ornament such as the vermiform grooves illustrated above. Similar grooving is known on some lungfish skulls, such as *Ctenodus interruptus* (Watson & Gill, 1923; Sharp & Clack, 2013) but it is not combined with an otherwise pustular surface. The ornament type in the postparietals illustrated here was confirmed as rhizodont by J. Jeffery (2018) from his personal observations, but has never been illustrated in the literature.

Unknown morphologies have also been uncovered. One of our specimens is a particular puzzle and does not fit easily into known categories, and a second, a rhizodont pterygoid, could challenge information from the published literature. These discoveries point out how little of the earliest Carboniferous is known in terms of vertebrate fossils, and isolated elements emphasize the level of our ignorance.

### Microfossil assemblage

Given their limited representation in the macrofossil assemblage, an unexpected actinopterygian abundance is revealed in the micropaleontological sample. In total, actinopterygian microfossils comprise 55.3 specimens per gram, while rhizodont microfossils only 11.5 per gram (SI1). Actinopterygian microfossils are more common than rhizodont microfossils in the 250 and 125 μm size fractions (Fig. 21). This would imply that actinopterygians are more abundant than rhizodonts, surprising given their difference in size—rhizodonts are approximately 10 times larger than actinopterygians, and so could be expected to produce a lot more microfossil fragments. The greater fragility and breakage of actinopterygian scales compared to rhizodont scales counters this interpretation, along with the taphonomic concentration or winnowing of a certain size of material during transport. However, the teeth of actinopterygians and rhizodonts recovered in this study are of a similar size and there are approximately ten times the number of actinopterygian teeth (excluding pharyngeal teeth) than rhizodont teeth. This indicates that the relative abundance of actinopterygians was probably higher than that of rhizodonts.

### Interpretation and environment of Bed 383

The six packages are interpreted as channelized river deposits, the channel forms being clear from the aerial views. Packages 1–3 are laterally adjacent channels and each one is successively younger, potentially representing shallow river channels laterally migrating through time (Fig. 2A). The dolostones overlying these three packages indicate a time during which a standing body of water developed, probably a lake (Bennett et al., 2016). Package 4 represents the return of a river system to this area, with the associated erosional relief suggesting a more significant system. Sediment deposited in this channel was partially eroded, but package 5 may represent a phase of channel abandonment and the deposition of fine-grained sediments. The youngest, package 6, has the greatest erosional relief. Only one side of the channel is exposed, therefore, the large-scale stratification could represent bar progradation or lateral migration of the channel itself.

The localized conglomerate probably represents deposition in the deepest part of this channel (package 6) through bedload processes. The sedimentology of the conglomerate lag (with clasts of red and gray siltstone) indicate that floodplain sediments were incorporated, including clasts of sandy siltstones (Bennett et al., 2016) and paleosols (Kearsey et al., 2016). Erosion by these river systems of the surrounding vegetated floodplains is clear from the erosional relief observed at this location. Several of the specimens show evidence of abrasion from rolling, and most are chaotically distributed in the matrix. The iron oxide staining of many rhizodont scales indicates that some rhizodont carcases may have been subject to subaerial exposure. Staining may have occurred during the desiccation of a floodplain lake and oxidation of fossil material, prior to the incorporation of these sediments into the river system.

The presence of ichnofauna, rare scolecodonts, orthocones, and evaporites (gypsum and anhydrite) within the Ballagan Formation provide evidence that the floodplain was subject to occasional marine water input (Bennett et al., 2016; Millward et al., 2018a, 2018b). The *Chondrites* in dolostones below Bed 383 has been interpreted as short-lived colonization of coastal lake sediments by opportunistic organisms that were transported from shallow marine environments during storm events (Bennett et al., 2016). These *Chondrite*s-bioturbated dolostones suggest the development of a significant floodplain lake, prior to the fluvial systems (packages 4–6) being reestablished. This is consistent with the complex coastal to alluvial paleoenvironment that characterizes the Ballagan Formation. Dolostone and evaporite clasts have not been incorporated into the conglomerate. Xenacanths are often associated with freshwater deposits, and Carboniferous elasmobranchs, ctenacanths, and *Ageleodus* are also interpreted as having a euryhaline salinity tolerance and occur in a range of marine to continental environments (Carpenter et al., 2014). Elasmobranchs are more commonly associated with freshwater sedimentary deposits than contemporaneous holocephalan chondrichthyans (Friedman & Sallan, 2012).

The presence of plant and charcoal debris confirm that vegetated floodplains were eroded and were subject to fire. However, other evidence for subaerial floodplain ecosystems, for example, myriapods (Ross et al., 2018), are absent. The bedload transport process is likely to have broken and winnowed away arthropod cuticle and the fragile calcite shells of ostracods and bivalves, which are more common within floodplain facies (Bennett et al., 2016). The scarcity of tetrapod and dipnoan microfossil material may again reflect taphonomic processes related to the transport, concentration and deposition of fossil material. The presence of charcoal may indicate forest fires that increased the potential for erosion of the floodplain.

Burnmouth is a locality well known for the occurrence of anatomically preserved plants that were described in several papers by Albert Long (see Scott, Galtier & Clayton, 1984). The majority of the plants described were from dolostone facies of presumed late Tournaisian age obtained from loose pebbles. Scott, Galtier & Clayton (1984) reported in situ plants from nearby localities such as the shore section at Partanhall, Burnmouth (NT959614) and Scott & Rex (1987) illustrated a permineralized *Stauropteris berwickensis* in a silty-wackstone horizon that also contained “*Lepidodendron*” *calamopsoides* axes together with fish bones and ostracods. The flora described from loose blocks included a range of lycopsids, pteridosperms, and ferns. A similar flora was described in dolostone facies from the River Whiteadder, for example, at Edrom (Scott, Galtier & Clayton, 1984; Scott & Rex, 1987). In addition to permineralized plants, some preserved as charcoal were also recovered (Scott & Rex, 1987). Compression floras, together with permineralized plants and charcoal were also reported from Foulden, about four miles (6.4 km) from Burnmouth and also of Tournaisian age (Scott & Meyer-Berthaud, 1985; Scott & Rex, 1987; Scott & Galtier, 1996; Scott & Glasspool, 2006).

Fire has been an important influence on the Earth system throughout history (Scott, 2000, 2018). For there to be fires, there needs to be not only fuel to burn, and an ignition source (generally lightning) but also an atmospheric oxygen level of over 17% (Scott, 2010; Scott et al., 2014). The occurrence of charcoal in the sediments of this conglomerate allows two immediate conclusions. The first is that the oxygen level in the atmosphere was at least above 17% corroborating the hypothesis that the Tournaisian stage was not one subject to lower oxygen levels, and may even have been above the modern level of 21% (Glasspool et al., 2015; Rimmer et al., 2015). Secondly, there is evidence of wildfire that affected the floodplains. However, the occurrence of charcoal in the sediments offers a further possibility. If there are rainstorms following a wildfire then the area of the fire can experience postfire erosion (Moody & Martin, 2001; Cannon, 2001). This happens when the roots of the plants have been killed and the sudden influx of water leads to increased erosion and movement of both the sediment and plant material (as well as animal material on the soil surface). This slurry may move very quickly (Scott, 2010) and form almost instantaneous alluvial fans away from the place of the wildfire (Moody & Martin, 2001; Scott et al., 2014). Such sediment may be either be transported into a river or lake system directly (as following the frequent Yellowstone fires) (Meyer & Pierce, 2003; Pierce, Meyer & Jull, 2004) or else the sediments are quickly eroded by fluvial processes such as occurred in the Buffalo Creek fires of 1996 (Moody & Martin, 2001, 2009). Postfire erosion/depositional systems have only recently been recognized in the fossil record. Extensive sediment pulses with charcoal have been described from the Early Carboniferous (Mississippian) of Ireland where there has been a sudden influx of sediment into a marine near-shore environment with consequences for the fauna living there (Nichols & Jones, 1992; Falcon-Lang, 1998). The occurrence of vertebrates that have been caught up in such postfire flood events have also been described in the Triassic (Havlik et al., 2013) and Cretaceous by several authors (Brown, Collinson & Scott, 2013; Muir, Bordy & Prevec, 2015).

The vertebrate remains alongside the charcoal, and the greater depth of erosion observed associated with package 6 suggest that postfire erosion/deposition may have played a role in its formation. Charcoal often floats and takes time to settle in a water column, depending on size, type of organ, and temperature of formation and hence can travel considerable distances by water transport (Nichols et al., 2000; Scott, 2010). Charcoal is often deposited by currents of a certain velocity where fragments are also well sorted (Nichols et al., 2000). The occurrence of charcoal and uncharred plants with siltstone and sand-sized grains alongside larger pebbles (paleosols and sandy siltstone) could suggest a more chaotic flow and rapid deposition for at least this part of the bed. However, the stratification in the channel fill above the conglomerate indicates large-scale bed-forms and sustained period of sandstone deposition. The widespread occurrence of charcoal suggests that fire appears to have been a regular occurrence through the Mississippian of Berwickshire and across the south of Scotland and to have had an impact on both the vegetation and the landscape (Scott, 1988; Brown, Scott & Jones, 1994; Scott & Glasspool, 2006).

## Conclusions

This study has described and interpreted the fauna of an Early Carboniferous river system and aspects of its surroundings and environment. It has provided further evidence to demonstrate that the Tournaisian stage of the Early Carboniferous was by no means a depauperate time in the evolution of vertebrate life on land. Neither was it a time of low oxygen concentration. Instead, the richness of a single small channel-fill conglomerate demonstrates the diversity of vertebrate taxa and their potential abundance in previously understudied Tournaisian nonmarine fossil localities. It also shows that large-bodied taxa, in some cases larger than those from later in the Carboniferous, lived in the Tournaisian, about nine Myr after the end of the Devonian. Thus large forms appear to have recovered more quickly following the Hangenberg extinction event than previous estimates of up to 36 Myr (Sallan & Gallimberti, 2015) had suggested. The existence of the so-called “Romer’s Gap” thus largely resulted from lack of exploration of such localities, and efforts should be made to target equivalent localities in other parts of the world.

## Supplemental Information

10.7717/peerj.5972/supp-1 Supplemental Information 1 Microfossil data. A list of micro fossil specimens used in the faunal analysis.

## Additional Information and Declarations

### Data Availability

The following information was supplied regarding data availability: Dryad: DOI 10.5061/dryad.2mg27sh
