# Supplementary material for: A Tournaisian (earliest Carboniferous) conglomerate-preserved non-marine faunal assemblage and its environmental and sedimentological context
Source: PeerJ. 2019 Jan 3;6:e5972. doi: 10.7717/peerj.5972 (PMC6321757; doi:10.7717/peerj.5972)
Supplement: Supplemental Information 1 — A list of micro fossil specimens used in the faunal analysis. [file peerj-07-5972-s001.docx]

|  |  |  |  |  |
| --- | --- | --- | --- | --- |
| **Fraction (μm)** | **Material** | **Description** | **Count** |  |
| 1000 | Rhizodont scales | fibrous, cream coloured, shiny exterior, tubercules or ridges on interior, some specimens with red staining (n = 23) | 67 |  |
|  | Rhizodont dermal bone | thick, flat, brown coloured bone fragments, one surface is flat, the other has irregular tubercule shapes | 1 |  |
|  | Rhizodont tooth | wide with striations on surface | 1 |  |
|  | Actinopt. scale | diamond shaped, striated grooves, shiny, brown to beige colour | 11 |  |
|  | Actinopt. dermal bone | thick, flat, brown coloured bone fragments, one surface is flat, the other has irregular tubercule shapes | 9 |  |
|  | Actinopt. bone | small tubular lepidotrichia bones | 2 |  |
|  | Chondrichthyan scale | elasmobranch indet. (n = 6), hybodont (7), ?ctenacanth (1) and indet. chondrichthyan denticles | 22 |  |
|  | *Ageleodus* tooth | specimens with a complete root and broken teeth cusps | 2 |  |
|  | Xenacanth tooth | three pronged tooth | 1 |  |
|  | Dipnoan toothplate | flat plate with different sized tubercules, some are broken | 1 |  |
|  | Indet. fish bone | phosphatic bone fragments, various shapes, colours and textures, indet. | 56 |  |
|  | Indet. fish scale | phosphatic fish scale fragments, various shapes, colours and textures, indet. | 29 |  |
|  | White scale indet. | thick fragments with elongate grooves and pits | 5 |  |
|  | Charcoal | black coloured, fibrous, brittle, with preserved cellular internal structures | 1 |  |
|  |  | Total | 208 |  |
| 425 | Rhizodont scales | fibrous, cream coloured, shiny exterior, tubercules or ridges on interior, some specimens with red staining (n = 52) | 359 |  |
|  | Actinopt. scale | diamond shaped, striated grooves, shiny, brown to beige colour | 165 |  |
|  | Actinopt. dermal bone | chunky fragments, flat on one side, bumpy with nodules on the other side, brown | 8 |  |
|  | Actinopt. teeth | tapering, with a transparent tip (3) and pharangeal teeth (2) | 5 |  |
|  | Actinopt. bone | small tubular lepidotrichia bones | 67 |  |
|  | Chondrichthyan scale | elasmobranch indet. (n = 34), hybodont (21), ?ctenacanth (4) and indet. chondrichthyan denticles | 95 |  |
|  | *Ageleodus* tooth | specimen with a complete base and broken teeth cusps | 1 |  |
|  | Indet. fish bone | phosphatic bone fragments, various shapes, colours and textures, indet. | 386 |  |
|  | Indet. fish scale | phosphatic fish scale fragments, various shapes, colours and textures, indet. | 154 |  |
|  | White scale indet. | thick fragments with elongate grooves and pits | 19 |  |
|  | Teeth indet. | squat shape | 2 |  |
|  | charcoal | black coloured, fibrous, brittle, with preserved cellular internal structures | 1 |  |
|  |  | Total | 1262 |  |
| 250 | Rhizodont scales | fibrous, cream coloured, shiny exterior, tubercules or ridges on interior, some specimens with red staining (n = 13) | 174 |  |
|  | Rhizodont teeth | curved fragments with striations on surface | 2 |  |
|  | Actinopt. scale | diamond shaped, striated grooves, shiny, brown to beige colour | 332 |  |
|  | Actinopt. teeth | tapering, with a transparent tip (10) and pharangeal teeth (20) | 30 |  |
|  | Actinopt. bone | small tubular lepidotrichia bones | 83 |  |
|  | Chondrichthyan scale | elasmobranch indet. (n = 3) and indet. broken denticle fragments | 10 |  |
|  | Indet. fish bone | phosphatic bone fragments, various shapes, colours and textures, indet. | 234 |  |
|  | Indet. fish scale | phosphatic fish scale fragments, various shapes, colours and textures, indet. | 790 |  |
|  | charcoal | black coloured, fibrous, brittle, with preserved cellular internal structures | 1 |  |
|  |  | Total | 1656 |  |
| 125 | Rhizodont scales | fibrous, cream coloured, shiny exterior, tubercules or ridges on interior | 35 |  |
|  | Actinopt. scale | diamond shaped, striated grooves, shiny, brown to beige colour | 2264 |  |
|  | Actinopt. teeth | tapering, with a transparent tip (17) and pharangeal teeth (70) | 87 |  |
|  | Actinopt. bone | small tubular lepidotrichia bones | 19 |  |
|  | Chondrichthyan scale | indet. broken denticle fragments | 13 |  |
|  | Indet. fish bone | phosphatic bone fragments, various shapes, colours and textures, indet. | 33 |  |
|  | Indet. fish scale | phosphatic fish scale fragments, various shapes, colours and textures, indet. | 86 |  |
|  | charcoal | black coloured, fibrous, brittle, with preserved cellular internal structures | 31 |  |
|  |  | Total | 2568 |  |
|  |  |  |  | **microfossils/g** |
| Total | Rhizodont |  | 639 | 11.5 |
|  | Actinopt. |  | 3082 | 55.3 |
|  | Chondrichthyan |  | 144 | 2.6 |
|  | Dipnoan |  | 1 | 0.0 |
|  | Fish indet. |  | 1794 | 32.2 |
|  | Charcoal |  | 34 | 0.6 |
|  |  |  |  |  |
